# Squeezing, trisqueezing and quadsqueezing in a hybrid oscillator–spin system

**DOI:** 10.1038/s41567-026-03222-6

**Published:** 2026-05-01

**Authors:** O. Băzăvan, S. Saner, D. J. Webb, E. M. Ainley, P. Drmota, D. P. Nadlinger, G. Araneda, D. M. Lucas, C. J. Ballance, R. Srinivas

**Affiliations:** Department of Physics, University of Oxford, Clarendon Laboratory, Oxford, UK

**Keywords:** Quantum information, Quantum simulation, Atomic and molecular interactions with photons, Quantum metrology

## Abstract

Quantum harmonic oscillators model phenomena from electromagnetic fields to molecular vibrations, with excitations represented by bosons such as photons or phonons. Linear interactions that create or annihilate single bosons generate coherent states of light or motion. Introducing higher-order nonlinear interactions produces richer quantum behaviour: second-order interactions enable squeezing, whereas higher-order interactions generate non-Gaussian states useful for continuous-variable quantum computation. However, such interactions are usually weak or require specialized hardware. Hybrid systems, where a linear interaction couples an oscillator to a spin, offer an alternative. Here we combine two spin-dependent linear bosonic interactions to implement up to fourth-order nonlinear bosonic interactions in a single trapped ion, focusing on generalized squeezing. We demonstrate and characterize squeezing, trisqueezing and quadsqueezing; reconstruct the Wigner functions of the resulting states; and achieve quadsqueezing over 100 times faster than conventional methods. The approach has no fundamental limit on the interaction order and applies to any platform supporting spin-dependent linear interactions.

## Abstract (web summary)

Higher-order interactions in quantum harmonic oscillator systems can result in useful effects, but they are hard to engineer. An experiment on a single trapped ion now demonstrates how spin can mediate higher-order nonlinear bosonic interactions.

## Main

Nonlinear processes and interactions in quantum harmonic oscillators are ubiquitous in various technological and scientific applications, ranging from frequency conversion^1^ and nonlinear spectroscopy^2^ to the creation of non-classical states like entangled photon pairs^3^ and squeezed states^4^. Squeezed states, which are generated by second-order bosonic processes, reduce the uncertainty in one observable, such as position, whereas increasing it in its conjugate, that is, momentum^5^. Such states have been used for enhancing the sensitivity of gravitational-wave detectors^6^, microscopy^7^ and the measurement of small electric fields^8^. In contrast to conventional squeezing, which is Gaussian and can be efficiently simulated classically^9^, higher-order interactions are no longer Gaussian. Consequently, these higher-order interactions serve as a resource for the real-time quantum simulation of interacting boson models^10–12^, with the potential to surpass the capabilities of classical hardware. Non-Gaussian operations, such as the third-order generalized squeezing interaction^13^, or trisqueezing, are also essential for continuous-variable quantum computation. Together with Gaussian operations, such as displacement and squeezing, they enable computational universality and error correction^14–18^. Aside from being non-Gaussian, the resulting states from these interactions are also of foundational interest in quantum mechanics, as they can exhibit non-classical properties such as Wigner negativity^19–21^.

However, realizing these nonlinear bosonic interactions faster than decoherence mechanisms has posed experimental challenges, especially as the interaction strength diminishes with increasing order. Generating any one of these interactions typically requires careful hardware considerations such as specifically tailored ion trap geometries^22^ or the design of superconducting microwave circuits^23,24^. For example, although the squeezing of a harmonic oscillator has been demonstrated using electromagnetic fields^25^, mechanical oscillators^26^ and trapped ions^27^, trisqueezing has only recently been demonstrated by refs. ^28,29^ in superconducting microwave circuits. Engineering higher than third-order bosonic interactions has, so far, been an outstanding challenge.

Instead of creating direct bosonic interactions, hybrid oscillator–spin systems offer an additional degree of freedom, which can be used to mediate effective interactions. In such systems, the oscillator can be coupled to the spin via a spin-dependent interaction that is linear in the bosonic mode. These interactions are readily available in a variety of platforms, ranging from trapped ions^30^, atoms^31^ and superconducting qubits^32^ to diamond colour centres^33^, and used extensively to realize boson-mediated spin–spin entanglement that overcomes the intrinsically weak spin–spin interactions^34–36^. Here, following the proposal in ref. ^37^, we instead use spin to mediate bosonic interactions. Focusing on generalized squeezing, we drive two of these linear spin-dependent interactions concurrently to demonstrate up to fourth-order bosonic interactions using a single trapped ion whose motion is a harmonic oscillator that can be coupled to its internal spin states. In particular, we use the same two linear interactions to create squeezing, trisqueezing and quadsqueezing by simply adjusting the interaction frequency.

**Fig. 1:**
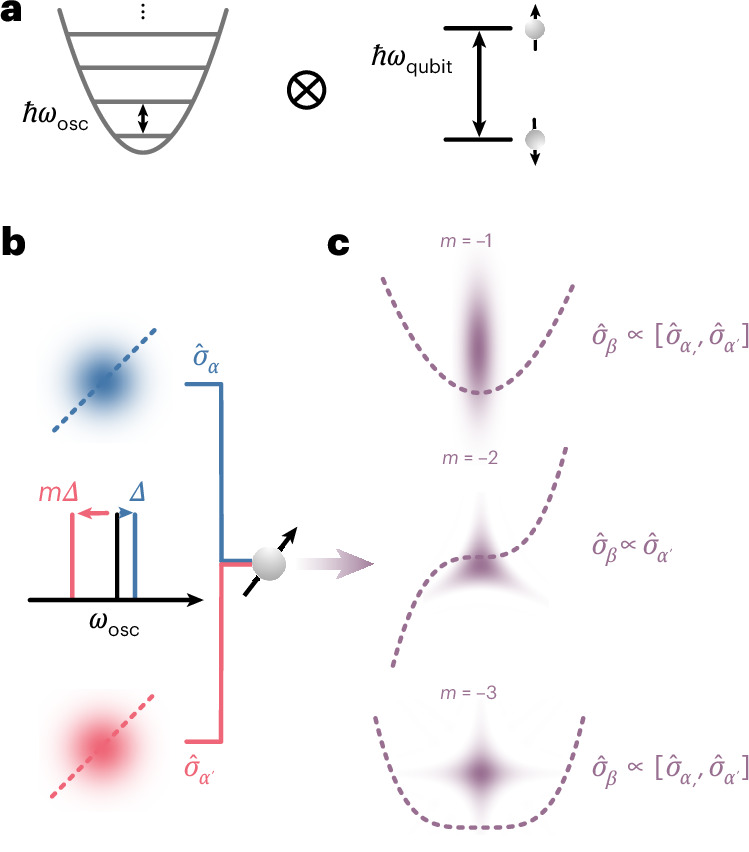
Conceptual illustration of spin-mediated nonlinear interactions. **a**, Hybrid oscillator–spin system. The protocol requires a quantum harmonic oscillator with energy splitting *ℏω*_osc_ (left) coupled to a spin system with energy splitting *ℏω*_qubit_ (right). **b**, Frequency settings for spin-dependent linear interactions. We apply two SDFs that are detuned from the oscillator motion frequency *ω*_osc_ by Δ and *m*Δ, where *m* is an integer. These interactions are linear and cause a spin-dependent displacement. We set the spin components of these forces ${\widehat{\sigma }}_{\alpha }$ and ${\widehat{\sigma }}_{{\alpha }^{{\prime} }}$ such that they do not commute, that is, $[{\widehat{\sigma }}_{\alpha },{\widehat{\sigma }}_{\alpha }^{{\prime} }]\ne 0$. We show the Wigner functions of the coherent states (blue and red blobs) that would be generated by the effective potential of the linear interactions (blue and red dashed lines). **c**, Generation of nonlinear interactions. By adjusting the relative detunings of the linear interactions, and hence *m*, we can drive arbitrary nonlinear interactions. Setting *m* = −1 gives rise to squeezing $\sim ({\widehat{a}}^{\dagger 2}+{\widehat{a}}^{2})$; *m* = −2, to trisqueezing $\sim ({\widehat{a}}^{\dagger 3}+{\widehat{a}}^{3})$; and *m* = −3, to quadsqueezing $\sim ({\widehat{a}}^{\dagger 4}+{\widehat{a}}^{4})$. The purple dashed lines indicate the effective potential for nonlinear interactions that are proportional to ${({\widehat{a}}^{\dagger }+\widehat{a})}^{n}$; by setting *m* = 1 − *n*, we can select the terms in the expansion of this potential that correspond to generalized squeezing interactions. The Wigner functions of the corresponding generalized squeezed states are overlaid on top in purple.

To elucidate how we generate these *n*th-order interactions, we first consider the quantum harmonic oscillator, which can be described by the operators ${\widehat{a}}^{\dagger }$ and $\widehat{a}$ that create and annihilate a boson, respectively. In hybrid systems (Fig. 1a), this oscillator can be coupled to a spin via a spin-dependent force (SDF) described by the interaction Hamiltonian

1 $${\widehat{H}}_{\mathrm{SDF}}=\frac{\hslash {\varOmega }_{\alpha }}{2}{\widehat{\sigma }}_{\alpha }\left(\widehat{a}{{\rm{e}}}^{-{\rm{i}}(\Delta t+{\phi }_{\alpha })}+{\widehat{a}}^{\dagger }{{\rm{e}}}^{{\rm{i}}(\Delta t+{\phi }_{\alpha })}\right),$$

which is linear in ${\widehat{a}}^{\dagger }$ and $\widehat{a}$. This type of interaction can be generated in many systems such as photons in a microwave cavity coupled to a superconducting qubit^32^, or phonons coupled to the internal spin state of trapped ions^30^. The coupling to the spin is described by the Hermitian operator ${\widehat{\sigma }}_{\alpha }$, which is a linear combination of the Pauli operators ${\widehat{\sigma }}_{x,y,z}$. The SDF results in a displacement of the harmonic oscillator state, conditioned on the spin state. This displacement depends on the interaction strength *Ω*_*α*_, as well as Δ and *ϕ*_*α*_, which are the detuning and phase, respectively, of the SDF relative to the harmonic oscillator with frequency *ω*_osc_.

The nonlinear spin-dependent interactions we seek to generate are the generalized squeezing interactions^13^ described by

2 $${\widehat{H}}_{{\rm{NL}}}=\frac{\hslash {\varOmega }_{n}}{2}{\widehat{\sigma }}_{\beta }({\widehat{a}}^{n}{{\rm{e}}}^{-{\rm{i}}\theta }+{\widehat{a}}^{\dagger n}{{\rm{e}}}^{{\rm{i}}\theta }),$$

where *n* is the order of the interaction, *Ω*_*n*_ is its strength and *θ* is the axis of the interaction. Here ${\widehat{\sigma }}_{\beta }$ is a Hermitian spin operator, defined similarly to ${\widehat{\sigma }}_{\alpha }$ as a linear combination of ${\widehat{\sigma }}_{x,y,z}$. For *n* = 2, 3, 4, this corresponds to spin-dependent squeezing, trisqueezing and quadsqueezing, respectively. Applying the Hamiltonian in equation (2) for a duration *t*_sqz_ generates *n*th-order squeezed states characterized by the squeezing parameter *r* = *Ω*_*n*_*t*_sqz_. Using conventional techniques, the higher the order of the interaction, the more demanding it is to generate. For example, in trapped ions, these interactions are conventionally driven by higher-order spatial derivatives of the electromagnetic field^27,38^, where *Ω*_*n*_ varies with *η*^*n*^. The Lamb–Dicke parameter *η* corresponds to the ratio of the ground-state extent of the ion (~10 nm) to the wavelength of the driving field (~500 nm). Thus, *η* is usually small and every subsequent order is weaker by more than an order of magnitude. This unfavourable scaling holds not only for trapped ions but also for other platforms such as superconducting circuits^39^.

Here we circumvent this scaling by instead combining two non-commuting SDFs, each of which is linear. Together, they generate a plethora of nonlinear interactions with different resonance conditions, as proposed in ref. ^37^ (Fig. 1b,c). The interaction is then described by

3 $$\begin{array}{rcl}\widehat{H} & = & \frac{\hslash {\varOmega}_{\alpha}}{2}{\widehat{\sigma}}_{\alpha}(\widehat{a}{{\rm{e}}}^{-{\rm{i}}\Delta t}+{\widehat{a}}^{\dagger}{{\rm{e}}}^{{\rm{i}}\Delta t})\\ & & +\frac{\hslash {\Omega}_{{\alpha}^{{\prime}}}}{2}{\widehat{\sigma}}_{{\alpha}^{{\prime} }}(\widehat{a}{{\rm{e}}}^{-{\rm{i}}(m\Delta t+{\phi }_{{\alpha}^{{\prime} }})}+{\widehat{a}}^{\dagger}{{\rm{e}}}^{{\rm{i}}(m\Delta t+{\phi}_{{\alpha}^{{\prime} }})}),\end{array}$$

where Δ and *m*Δ (*m* is an integer) are the detunings from *ω*_osc_. Without loss of generality, we set *ϕ*_*α*_ = 0. If the spin components of the two forces do not commute, that is, $[{\widehat{\sigma }}_{\alpha },{\widehat{\sigma }}_{{\alpha }^{{\prime} }}]\ne 0$, we can choose *m* = 1 − *n* to satisfy the resonance condition for creating effective interactions corresponding to equation (2) (this is true up to a phase redefinition for even *n*; Supplementary Information). For *m* = −1, −2, −3, we generate squeezing, trisqueezing and quadsqueezing interactions, respectively. The spin dependence ${\widehat{\sigma }}_{\beta }$ is given by the initial choice of ${\widehat{\sigma }}_{\alpha ,{\alpha }^{{\prime} }}$ and the desired squeezing order *n*. The even-order interactions have a spin dependence that follows ${\widehat{\sigma }}_{\beta }\propto [{\widehat{\sigma }}_{\alpha },{\widehat{\sigma }}_{{\alpha }^{{\prime} }}]$, whereas the odd orders follow ${\widehat{\sigma }}_{\beta }\propto {\widehat{\sigma }}_{{\alpha }^{{\prime} }}$. Hence, by being able to generate SDFs conditioned on any Pauli operator, the spin component of the nonlinear interaction can be arbitrarily chosen. The axis *θ* of the resulting interaction (equation (2)) can be controlled by adjusting the SDF phase ${\phi }_{{\alpha }^{{\prime} }}$. The strength of generalized squeezing *Ω*_*n*_ is proportional to ${\Omega }_{{\alpha }^{{\prime} }}{\Omega }_{\alpha }^{n-1}/{\Delta }^{n-1}$. Importantly, and contrary to previous implementations^27^, *Ω*_*n*_ can be made effectively linear with *η* for all orders *n* by appropriate choice of the detuning Δ, which is a free parameter in our scheme. Although both *Ω*_*α*_ and ${\varOmega }_{{\alpha }^{{\prime} }}$ scale as *η*, tuning Δ allows the overall scaling of *Ω*_*n*_ to remain effectively linear with *η*. Although *Ω*_*n*_ still decreases with increasing *n*, this method substantially enhances the effective interaction strength compared with direct driving of the *n*^th^-order sideband (Supplementary Fig. 1 and Supplementary Section VII).

We experimentally demonstrate these interactions on a trapped ^88^Sr^+^ ion in a three-dimensional radio-frequency Paul trap^40^. The ion vibrates in three dimensions; the harmonic oscillator used in this work is defined by the motional mode along the trap axis, with *ω*_osc_/2π ≈ 1.2 MHz (Fig. 1a). We initialize this oscillator close to the ground state with ${\bar{n}}_{{\rm{osc}}}=0.09(1)$. Aside from the motional degree of freedom, we use the $| 5{S}_{1/2},\,{m}_{j}=-\frac{1}{2}\rangle \equiv | \downarrow \rangle$ and $| 4{D}_{5/2},\,{m}_{j}=-\frac{3}{2}\rangle \equiv | \uparrow \rangle$ sublevels of the ion’s electronic structure to define our qubit, where *m*_*j*_ is the projection of the total angular momentum along the quantization axis defined by a 146-G static magnetic field.

For creating the nonlinear interactions, we use two SDFs, as described in equation (3), following the Mølmer–Sørensen-type scheme^36^. Each SDF requires a bichromatic field composed of two tones that are symmetrically detuned from the qubit transition *ω*_qubit_, driven by a 674-nm laser. If the tones are detuned by approximately ±*ω*_osc_, the spin component of the force is ${\widehat{\sigma }}_{\phi }=\cos \phi {\widehat{\sigma }}_{x}+\sin \phi {\widehat{\sigma }}_{y}$, where *ϕ* is given by the mean optical phase of the two tones at the position of the ion. Alternatively, we can obtain a ${\widehat{\sigma }}_{z}$ spin component by setting the detuning to be approximately ±*ω*_osc_/2 (refs. ^41,42^). We actively stabilize the optical phase between the laser beams that give rise to the SDFs to maintain their non-commuting relationship throughout the experiment. In our setup, the beam waist radius is 20 μm and the Lamb–Dicke parameter is *η* = 0.049(1). If the interaction SDF is in the ${\widehat{\sigma }}_{\phi }$ basis, then its strength is ${\varOmega }_{\alpha ,{\alpha }^{{\prime} }}/2{\rm{\pi }}\approx 4.6\,\,{\rm{kHz}}$ (laser power, 0.5 mW) or ~6.5 kHz (laser power, 1 mW). In the ${\widehat{\sigma }}_{z}$ basis, its strength is ~1.3 kHz (laser power, 1 mW). Moreover, to ensure that the effective Hamiltonian of the resulting nonlinear interactions tends to the ideal Hamiltonian in equation (2), we ramp the two bichromatic fields on and off with a sin^2^ pulse shape. The ramp duration *t*_ramp_ should be long compared with 2π/Δ. We characterize the oscillator states generated through the nonlinear interactions by applying a probe SDF on resonance with *ω*_osc_. The probe SDF is also created using an Mølmer–Sørensen scheme. We present complete details of the experimental setup in Supplementary Section IIA.

**Fig. 2:**
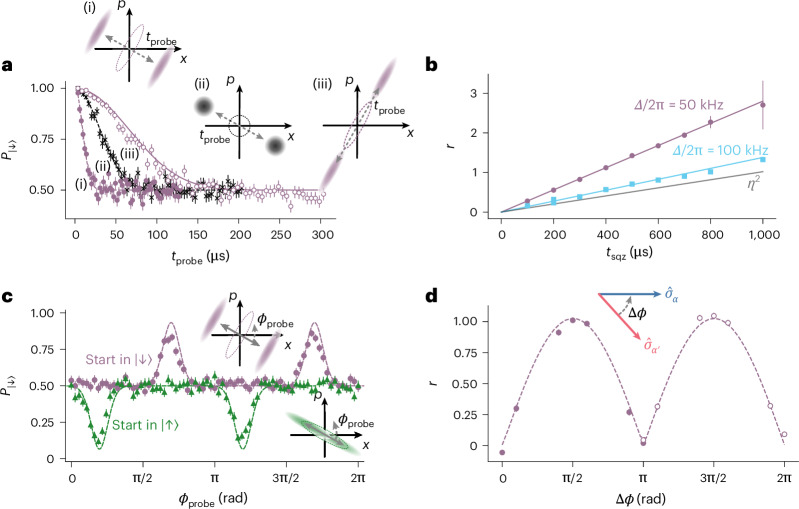
Characterization of the spin-dependent squeezing interaction. After applying the squeezing interaction, we use a probe SDF to map the oscillator state onto the spin population ${p}_{| \downarrow \rangle }$. Insets illustrate the action of the probe SDF on Wigner functions; the dashed ellipses indicate the pre-probe state. **a**, Inferring the squeezing parameter *r*. Varying the probe duration *t*_probe_ yields a spin-dependent displacement that separates the wavefunction (insets). The probe is applied along the two principal axes of a squeezed state (i) and (iii) and to a near-ground-state thermal state (ii). We obtain *r* = 1.09(4) by fitting (i) and ((ii); dashed lines). For splitting about the anti-squeezed axis (iii), we plot a numerical simulation (solid line) including motional decoherence. **b**, Detuning dependence. We plot *r* versus *t*_sqz_ for Δ/2π = 50 kHz and Δ/2π = 100 kHz. Theory (solid purple/cyan) follows *r* = *Ω*_2_*t*_sqz_. The solid grey line shows the *r* expected from driving the second-order spatial derivative of the field at equal laser power. **c**, Spin dependence. With a fixed probe duration, we vary its phase *ϕ*_probe_. Fits (dashed) show peaks/dips when the probe aligns with the anti-squeezed axis; flipping the initial spin from $| \downarrow \rangle$ to $| \uparrow \rangle$ shifts the pattern by π/2. **d**, Non-commutativity of interaction SDFs. Two interaction SDFs with bases ${\widehat{\sigma }}_{\phi }$ and ${\widehat{\sigma }}_{\phi +\Delta \phi }$ yield *r*(Δ*ϕ*); commuting cases (Δ*ϕ* = 0, π, 2π) give *r* ≈ 0, whereas non-commuting (π/2, 3π/2) maximize squeezing. Data are fit with $A| \sin \Delta \phi |$ (dashed). Marker fill indicates probe-phase setting. **a** and **c** show 68% confidence intervals from shot noise with 300 shots per point and centre equal to the measured ${P}_{| \downarrow \rangle }$; **b** and **d** show 68% confidence intervals derived from the fit and centre equal to the fitted *r*. The error bars are occasionally smaller than the marker size. Source data

We first use this technique to generate spin-dependent squeezing (*n* = 2 in equation (2)) and verify the key characteristics of this interaction family: magnitude, spin dependence and non-commutativity (Fig. 2). These interactions are also unitary, which we investigate in Supplementary Section V. We set the detunings of the SDFs to be Δ and −Δ, respectively, that is *m* = −1. The spin components of the two SDFs are set to ${\widehat{\sigma }}_{\alpha }={\widehat{\sigma }}_{\phi }$ and ${\widehat{\sigma }}_{{\alpha }^{{\prime} }}={\widehat{\sigma }}_{\phi +{\rm{\pi }}/2}$, respectively. Thus, the spin basis of the squeezing is $[{\widehat{\sigma }}_{\alpha },{\widehat{\sigma }}_{{\alpha }^{{\prime} }}]\propto {\widehat{\sigma }}_{z}$. If we start in $| \downarrow \rangle$ or $| \uparrow \rangle$ (eigenstates of ${\widehat{\sigma }}_{z}$), the spin component remains unchanged and the squeezing axis depends on the spin state. Once the squeezed state is created, we apply a probe SDF with the spin component in the ${\widehat{\sigma }}_{x}$ basis with eigenstates $| \pm \rangle =(| \uparrow \rangle \pm | \downarrow \rangle )/\sqrt{2}$. Hence, the probe SDF displaces the $| +\rangle$ and $| -\rangle$ components of the resulting state in opposite directions^43^ (Fig. 2a, insets). The overlap of the two parts of the harmonic oscillator wavefunction is mapped onto the spin, whose state probability ${P}_{| \downarrow \rangle }$ is measured by the fluorescence read-out. We apply the probe SDF for variable durations *t*_probe_; as *t*_probe_ increases, the overlap reduces and ${P}_{| \downarrow \rangle }\to 0.5$.

As shown in Fig. 2a, applying the probe along the squeezing axis (i) reduces the overlap faster than applying the probe orthogonal to the squeezing axis (anti-squeezed axis; (iii)). We determine the magnitude of the squeezing parameter^44^ *r* by fitting the splitting dynamics of a squeezed (i) and the initial thermal state with ${\bar{n}}_{{\rm{osc}}}=0.09(1)$ (ii), where the latter is used to calibrate the magnitude of the probe SDF. The inferred *r* = 1.09(4), equivalent to 9.5(3) dB of squeezing. Extracting *r* from (iii) using the analytic model underestimates the value of *r* due to motional decoherence, whose effect is more apparent in this case as it takes longer to reduce the overlap completely. Nonetheless, the resulting dynamics agree well with numerical simulations that incorporate the motional decoherence. The squeezed state considered here is created by using 0.5 mW for driving each interaction SDF, setting Δ/2π = 50 kHz and applying the interaction for a pulse duration of *t*_sqz_ = 400 μs with a ramp duration of *t*_ramp_ = 40 μs (all the pulse durations quoted in this text are measured at a full-width at half-maximum of the pulse shape; the ramp shape is $\sin {({\rm{\pi }}t/2{t}_{{\rm{ramp}}})}^{2}$ with a total rise time given by the ramp duration *t*_ramp_).

The squeezing parameter of the squeezed state is *r* = *Ω*_2_*t*_sqz_, where ${\varOmega }_{2}={\varOmega }_{\alpha }{\varOmega }_{{\alpha }^{{\prime} }}/\Delta$ following equation (2). We verify this dependence in Fig. 2b where we plot *r* as a function of *t*_sqz_ for Δ/2π = 50 kHz and Δ/2π = 100 kHz. The data agree well with the theory, calculated from independently measured values of ${\Omega }_{\alpha }\,\mathrm{and}\,{\Omega }_{{\alpha }^{{\prime} }}$, and we observe that the magnitude is inversely proportional to Δ. We compare the squeezing strength generated by our method to driving the interaction directly using the second-order spatial derivative of the field^27^. This interaction strength scales with *η*^2^ and the values were inferred by considering the same total power of 1 mW for both methods. This underscores that we can adjust the free parameter Δ in our method to achieve a higher coupling strength than driving the second-order interaction directly.

We next investigate the spin dependence of the interaction (Fig. 2c). The spin dependence of our interaction is in contrast to spin-independent squeezing achieved by modulating the confinement of the trapped ions^8,45,46^. We create squeezed states using the same parameters as those shown in Fig. 2a, and fix the probe SDF duration as *t*_probe_ = 53.6 μs. We scan the phase of the probe SDF *ϕ*_probe_ and measure ${P}_{| \downarrow \rangle }$. Changing this phase influences the direction about which we split the oscillator wavefunction (insets). The peaks and dips of the population correspond to splitting about the anti-squeezed axis and has a periodicity of π. There is a π/2 shift between the two curves as a result of squeezing about orthogonal axes in phase space introduced by the different spin-state settings (insets).

To generate this family of interactions, the spin components of the SDFs must be non-commuting. We explore this non-commutativity by varying the phase between the spin components of the two SDFs, that is, one of the forces is ${\widehat{\sigma }}_{\alpha }={\widehat{\sigma }}_{\phi }$ and the other is ${\widehat{\sigma }}_{{\alpha }^{{\prime} }}={\widehat{\sigma }}_{\phi +\Delta \phi }$. We measure *r* as a function of Δ*ϕ*, keeping the phase of the probe SDF constant. The squeezing parameter *r* varies as $\sin (\Delta \phi )$ following the commutator relationship $[{\widehat{\sigma }}_{\phi },{\widehat{\sigma }}_{\phi +\Delta \phi }]\propto \sin (\Delta \phi ){\widehat{\sigma }}_{z}$ (Fig. 2d). If the spin components commute, that is, Δ*ϕ* = 0, π and 2π, there is no squeezing, whereas for Δ*ϕ* = π/2 and 3π/2, the commutator of the spin components, and hence the squeezing, is maximized. When $\sin (\Delta \phi )$ becomes negative, that is, Δ*ϕ* > π, the axis of squeezing shifts by π/2; hence, we change the phase of the probe SDF to *ϕ*_probe_ + π/2 such that we always split about the squeezed axis.

So far, we have focused on squeezed states that have been explored in a variety of platforms. Moving to higher-order interactions, we reconstruct the Wigner quasiprobability function^47^ of the resulting quantum states to obtain their full description. Following ref. ^48^, we measure the complex-valued characteristic function $\chi (\beta )=\langle \widehat{{\mathcal{D}}}(\beta )\rangle$, where $\widehat{{D}}(\beta )={{\rm{e}}}^{\beta {\widehat{a}}^{\dagger }-{\beta }^{* }\widehat{a}}$ is the displacement operator and $\beta \in {\mathbb{C}}$ quantifies the displacement of the oscillator state in phase space. This measurement is an extension of the method discussed in Fig. 2, where we apply a probe SDF to split the oscillator wavefunction. Here we scan both *t*_probe_ and *ϕ*_probe_ to obtain the real and imaginary parts of the characteristic function, where $\beta \propto {t}_{\mathrm{probe}}\times {{\rm{e}}}^{{\rm{i}}{\phi }_{\mathrm{probe}}}$ (Supplementary Section VI). We then take the two-dimensional discrete Fourier transform of the measured characteristic function *χ*(*β*) to obtain the Wigner function *W*(*x*, *p*), where *x* and *p* are the position and momentum variables associated with the dimensionless position and momentum operators $\widehat{x}\,\mathrm{and}\,\widehat{p}$, respectively.

**Fig. 3:**
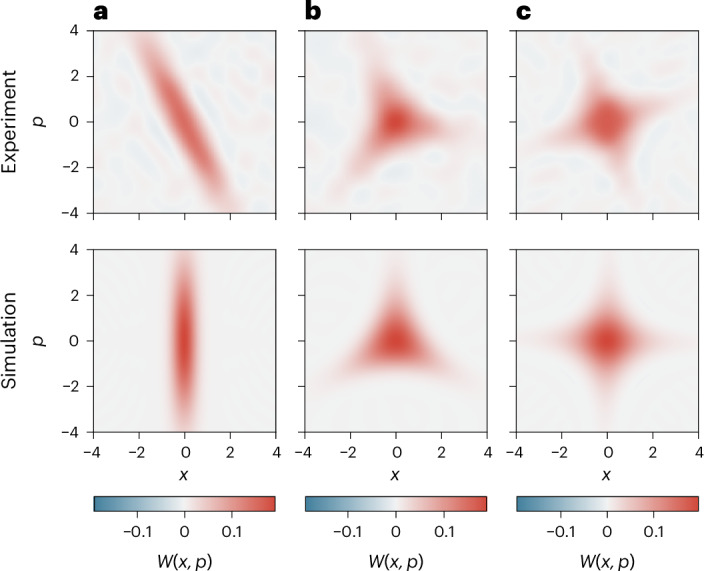
Wigner functions of generalized squeezed states. **a**, Squeezed state with *r* = 1.09(4). **b**, Trisqueezed state with *r*_3s_ = 0.19(1). **c**, Quadsqueezed state with *r*_4s_ = 0.054(5). In the top row, we show Wigner functions *W*(*x*, *p*) reconstructed from the experimental data, where *x* and *p* are the position and momentum variables associated with the dimensionless position and momentum operators $\widehat{x}\,\mathrm{and}\,\widehat{p}$, respectively. The Wigner function is inferred from the measured characteristic function of the oscillator state (see the main text). In the bottom row, we show Wigner functions of numerically simulated states with independently measured experimental parameters. The rotation observed compared with the simulation is due to a constant offset between the squeezing axis *θ* and the phase of the probing SDF *ϕ*_probe_. This offset can be calibrated out, if desired. Source data

We reconstruct the Wigner functions of experimentally implemented squeezed, trisqueezed and quadsqueezed states, and compare them with numerical simulations in which the experimental parameters were measured independently. Harnessing the versatility of our method, the trisqueezed and quadsqueezed states were created by simply changing the detuning *m*Δ. The spin dependence of all the interactions was controlled to be ${\widehat{\sigma }}_{z}$ and we initialize the spin in the $| \downarrow \rangle$ eigenstate. In Fig. 3a, we evaluate a squeezed state with *r* = 1.09(4), which is created using the same parameters as those shown in Fig. 2a.

To create the trisqueezed state (Fig. 3b), we set the detunings of the SDFs to be Δ and –2Δ, with Δ/2π = −25 kHz (we choose this negative detuning Δ/2π = −25 kHz to avoid off-resonantly driving an interaction corresponding to another motional mode of the ion). We apply the interaction for *t*_sqz_ = 600 μs, with *t*_ramp_ = 80 μs. We use a laser power of 1 mW per interaction SDF. We infer the trisqueezing parameter *r*_3s_ = *Ω*_3_*t*_sqz_ = 0.19(1) by assuming that the interaction strength follows the theory ${\varOmega }_{{\alpha }^{{\prime} }}{\varOmega }_{\alpha }^{2}/(2{\Delta }^{2})$ and comparing with simulation (Supplementary Section VIII). The basis of the trisqueezing interaction is given by $[{\widehat{\sigma }}_{\alpha },[{\widehat{\sigma }}_{\alpha },{\widehat{\sigma }}_{{\alpha }^{{\prime} }}]]$. Here we set the bases of the comprising interaction SDFs to ${\widehat{\sigma }}_{\alpha }={\widehat{\sigma }}_{\phi }$ and ${\widehat{\sigma }}_{{\alpha }^{{\prime} }}={\widehat{\sigma }}_{z}$ such that the effective interaction has a ${\widehat{\sigma }}_{z}$-spin component.

Due to the initially impure thermal state, it becomes challenging to observe Wigner negativity; however, the resulting Wigner function still displays a clear departure from a Gaussian profile, confirming the non-Gaussian character of the trisqueezed state^49^.

Last, we create quadsqueezed states (Fig. 3c) by setting the SDF detunings to be Δ and –3Δ, with Δ/2π = 25 kHz. We apply the interaction for *t*_sqz_ = 600 μs, with *t*_ramp_ = 80 μs. The laser power used is 1 mW per interaction SDF. Similar to the trisqueezed state, we determine the quadsqueezing parameter *r*_4s_ = *Ω*_4_*t*_sqz_ = 0.054(5). The spin basis of quadsqueezing is given by $[{\widehat{\sigma }}_{\alpha },[{\widehat{\sigma }}_{\alpha },[{\widehat{\sigma }}_{\alpha },{\widehat{\sigma }}_{{\alpha }^{{\prime} }}]]]$. Thus, choosing the basis of the comprising interaction SDFs to be ${\widehat{\sigma }}_{\alpha }={\widehat{\sigma }}_{\phi }$ and ${\widehat{\sigma }}_{{\alpha }^{{\prime} }}={\widehat{\sigma }}_{\phi +{\rm{\pi }}/2}$, we again achieve a ${\widehat{\sigma }}_{z}$ interaction. Similar to the trisqueezed state, non-Gaussianity in the quadsqueezed state is evident from the Wigner function’s shape, which deviates from a Gaussian profile. In Supplementary Section IX, we also show a quadsqueezed state created by increasing the power to 2 mW and decreasing the pulse duration to 400 μs, which exhibits Wigner negativity.

To our knowledge, this is the first implementation of trisqueezing in an atomic system and the first demonstration of fourth-order generalized squeezing across any platform. Our demonstration has only been possible because of the bosonic interactions mediated by the spin; the quadsqueezing interaction is more than 100 times stronger than an interaction derived from higher-order spatial derivatives of the driving field, assuming the same total laser power (Supplementary Section VII).

Overall, our work explores nonlinear bosonic interactions mediated by the spin in a hybrid oscillator–spin system by repurposing interactions readily available across various platforms. Using the spin to combine multiple linear bosonic interactions, our technique enabled us to demonstrate fourth-order nonlinear interactions without any limit on the achievable order. These interactions would have been otherwise-inaccessible using previous techniques. Further, the effective interactions are not limited to only generalized squeezing interactions, as shown in this work, but any nonlinear bosonic interaction comprising other combinations of the creation and annihilation operators. Our proof-of-principle demonstration used only a single motional mode of an ion coupled to two of its internal states. Both these quantum degrees of freedom can be explored further. First, our technique readily extends to multiple modes^37^ of a single ion or a larger crystal to generate interactions such as the beamsplitter^50–52^, two-mode squeezing^53^ or cross-Kerr couplings^54^. Such multimode interactions are essential for implementing a universal gate set for scalable continuous-variable quantum computing^14,16^. Second, the spin dependence of bosonic interactions creates the enticing possibility of performing midcircuit measurements on the spin to create resourceful quantum states^55–57^ for quantum simulation, metrology or error correction. These higher-order nonlinear interactions in the oscillator, conditioned on the spin, can also be used to generate new spin–spin interactions that go beyond those achieved with only second-order bosonic interactions^58^. Finally, our technique extends to boson-spin encodings that have recently gained attention as they are more natively suited to simulate various physical models^59^, boson Hubbard model in condensed matter^12^, quantum field theories in particle physics^10,11^ or molecular quantum effects^60,61^. These hybrid encodings enable computational protocols that are inherently more robust to errors^62^, as well as reducing the computational requirements for representing a boson in a collection of qubits. This reduction is particularly beneficial for practical applications involving near-term devices with limited circuit depths.

## Online content

Any methods, additional references, Nature Portfolio reporting summaries, source data, extended data, supplementary information, acknowledgements, peer review information; details of author contributions and competing interests; and statements of data and code availability are available at 10.1038/s41567-026-03222-6.

## Supplementary information

Supplementary Information Supplementary Sections I–IX, Figs. 1–11, Table I and Discussion.

## Source data

Source Data Fig. 2 Underlying experimental and numerical data used to generate Fig. 2a–d.

Source Data Fig. 3 Underlying experimental and numerical data used to generate Fig. 3.

## Data Availability

Source data are provided with this paper. All other data that support the plots within this paper and other findings of this study are available from the corresponding authors upon reasonable request.
