# Supplementary material for: Squeezing, trisqueezing and quadsqueezing in a hybrid oscillator–spin system
Source: Nat Phys. 2026 May 1;22(5):757–62. doi: 10.1038/s41567-026-03222-6 (PMC13183582; doi:10.1038/s41567-026-03222-6)
Supplement: Supplementary file 1 — Supplementary Sections I–IX, Figs. 1–11, Table I and Discussion. [file 41567_2026_3222_MOESM1_ESM.pdf]

nature physics

Supplementary information https://doi.org/10.1038/s41567-026-03222-6

Squeezing, trisqueezing and quadsqueezing in a hybrid oscillator–spin system

In the format provided by the authors and unedited

Supplementary Material

CONTENTS

I. Theory 9
A. Spin-mediated nonlinear bosonic interactions 9
B. Generating the spin-dependent forces 11

II. Experimental Considerations 12
A. Setup 12
B. Ramp 12

III. Numerical simulations 12

IV. Squeezed state characterisation 14

V. Unitarity of the interactions 15

VI. Wigner function 16

VII. Scaling of the nonlinear interaction strength 17

VIII. Comparison of ideal and effective generalised squeezing interactions 18

IX. Quadsqueezed state with Wigner negativity 18

References 19

I. THEORY

A. Spin-mediated nonlinear bosonic interactions

In this section, we discuss how we generate effective spin-dependent nonlinear bosonic interactions by applying spin-dependent interactions that are linear in the bosonic creation $\hat{a}^\dagger$ and annihilation $\hat{a}$ operators. The theory was developed in Ref. 37.

We use spin-dependent forces (SDFs) to generate the required linear interactions. An SDF is described by

$$\hat{H}_{\mathrm{SDF}} = \frac{\hbar\Omega_\alpha}{2}\hat{\sigma}_\alpha(\hat{a}e^{-i(\Delta t+\phi_\alpha)} + \hat{a}^\dagger e^{i(\Delta t+\phi_\alpha)}), \tag{4}$$

where Δ and ϕ_α are the detuning and phase of the SDF from the harmonic oscillator with frequency ω_{osc}, respectively. The basis of the spin conditioning $\hat{\sigma}_\alpha$ is a Hermitian linear combination of the Pauli spin-operators $\hat{\sigma}_{x,y,z}$. The strength of the interaction, Ω_α, is proportional to the Lamb-Dicke factor η. This expression is in the interaction picture with respect to the qubit frequency ω_{qubit}, and the oscillator frequency ω_{osc}, where we apply the rotating wave approximation (RWA) with respect to ω_{qubit} and ω_{osc}.

As discussed in the main text, the nonlinear interactions are created by applying two SDFs simultaneously, but with different detunings Δ and $m\Delta$ and bases $\hat{\sigma}_\alpha$ and $\hat{\sigma}_{\alpha'}$. The resulting interaction is

$$\begin{aligned}\hat{H} = {}& \frac{\hbar\Omega_\alpha}{2}\hat{\sigma}_\alpha(\hat{a}e^{-i(\Delta t+\phi_\alpha)} + \hat{a}^\dagger e^{i(\Delta t+\phi_\alpha)}) \\ &+ \frac{\hbar\Omega_{\alpha'}}{2}\hat{\sigma}_{\alpha'}(\hat{a}e^{-i(m\Delta t+\phi_{\alpha'})} + \hat{a}^\dagger e^{i(m\Delta t+\phi_{\alpha'})}),\end{aligned} \tag{5}$$

where we set $\phi_\alpha = 0$. To determine the dynamics of the two SDFs, we consider the resulting unitary time propagator found via the Magnus-expansion[37,66,67]

$$U(t) = \mathscr{T}\left(e^{\frac{-i}{\hbar}\int_0^t \hat{H}(t')dt'}\right) \tag{6}$$

$$\simeq \exp\left(\frac{-i}{\hbar}\int_0^t dt_1 H_1\right. \tag{7}$$

$$-\frac{1}{2\hbar^2}\int_0^t\int_0^{t_1} dt_1 dt_2 [H_1, H_2] \tag{8}$$

$$+\frac{i}{6\hbar^3}\int_0^t\int_0^{t_1}\int_0^{t_2} dt_1 dt_2 dt_3 \left([H_1,[H_2,H_3]] + [[H_1,H_2],H_3]\right) \tag{9}$$

$$\begin{aligned}&+\frac{1}{12\hbar^4}\int_0^t\int_0^{t_1}\int_0^{t_2}\int_0^{t_3} dt_1 dt_2 dt_3 dt_4 \\ &([[[H_1,H_2],H_3],H_4] + [H_1,[[H_2,H_3],H_4]] \\ &\left.+[H_1,[H_2,[H_3,H_4]]] + [H_2,[H_3,[H_4,H_1]]])\right),\end{aligned} \tag{10}$$

where $\mathscr{T}$ denotes the time-ordering operator and $\hat{H}_k \equiv \hat{H}(t_k)$ is the Hamiltonian describing the system at time t_k. The expansion is truncated at the 4th-order as opposed to 3rd-order in Ref. 37.

The first-order term in the Magnus expansion (Eq. (7)) leads to periodic displacements (i.e. loops) of the oscillator in phase space. For durations that are integer multiples of $2\pi/\Delta$, the oscillator state returns to its original position. The second term gives rise to a geometric phase, which underlies the effective spin-spin interactions[34–36] in trapped ions where the motion mediates the interaction. If $\hat{\sigma}_\alpha, \hat{\sigma}_{\alpha'}$ commute, i.e. $[\hat{\sigma}_\alpha, \hat{\sigma}_{\alpha'}] = 0$, the second-order term (Eq. (8)) is only a scalar corresponding to this geometric phase, causing all higher orders to vanish. However, if the bases of the SDFs are chosen such that $[\hat{\sigma}_\alpha, \hat{\sigma}_{\alpha'}] \neq 0$, we obtain the sought-after nonlinear interactions. In this case, the spin mediates the higher-order bosonic interactions. By choosing the correct integer setting for the detuning m, each term can be brought into resonance separately, i.e., have the leading contribution to the dynamics. This scheme can be applied to a single motional mode to produce, for example, generalised squeezing interactions or the parity operator (i.e., the Hamiltonian is $\propto \hat{a}^\dagger\hat{a}$). If, instead, we apply each SDF to a separate motional mode, we realise multi-mode couplings such as beam-splitter or two-mode squeezing. Here, we focus on the generalised squeezing interactions as described in Eq. (2). We experimentally demonstrate squeezing ($n = 2$), trisqueezing ($n = 3$) and quadsqueezing ($n = 4$). Squeezing originates from the second-order term (Eq. (8)) in the Mangus expansion by setting $m = -1$, trisqueezing from the third-order term (Eq. (9)) by setting $m = -2$, and quadsqueezing from the fourth-order term (Eq. (10)) by setting $m = -3$.

The squeezing and the quadsqueezing Hamiltonians are given by

$$\hat{H}^n_{\mathrm{eff,even}} = \frac{i\hbar\Omega_n}{2}\hat{\sigma}_\beta(-\hat{a}^n e^{-i\theta} + \hat{a}^{\dagger n}e^{i\theta}) \tag{11}$$

with $n = 2$ and $n = 4$, respectively. The trisqueezing interaction with $n = 3$ is

$$\hat{H}^n_{\mathrm{eff,odd}} = \frac{\hbar\Omega_n}{2}\hat{\sigma}_\beta(\hat{a}^n e^{-i\theta} + \hat{a}^{\dagger n}e^{i\theta}). \tag{12}$$

There is a $\pi/2$ phase difference in the motional phase (interaction axis) θ. These expressions are slightly different from those in the main text, where we omitted the change in the motional phase for simplicity in Eq. (2). Moreover, if we set $\phi_\alpha = 0$ (as done in the experiment), it follows that $\theta = \phi_{\alpha'}$.

The magnitudes of these interactions are given by

$$\Omega_{2,3,4} = \left\{\frac{\Omega_{\alpha'}\Omega_\alpha}{\Delta}, \frac{\Omega_{\alpha'}\Omega_\alpha^2}{2\Delta^2}, \frac{\Omega_{\alpha'}\Omega_\alpha^3}{8\Delta^3}\right\}. \tag{13}$$

The spin conditioning, which results from the nested commutators relationships, is given by

$$\hat{\sigma}_\beta \propto \begin{cases}[\hat{\sigma}_\alpha, \hat{\sigma}_{\alpha'}] & \text{if } n \bmod 2 = 0 \\ \hat{\sigma}_{\alpha'} & \text{otherwise.}\end{cases} \tag{14}$$

As introduced above, if the spin components of the SDFs do not commute, the Magnus expansion in Eq. (6) has an infinite number of terms. Thus, for certain resonance conditions, the dynamics of the Hamiltonian in Eq. (5) corresponds to that of the desired generalised squeezing Hamiltonian (Eq. (11), (12)) up to an error due to coherent spurious terms. As detailed in the theoretical proposal, Ref. 37,

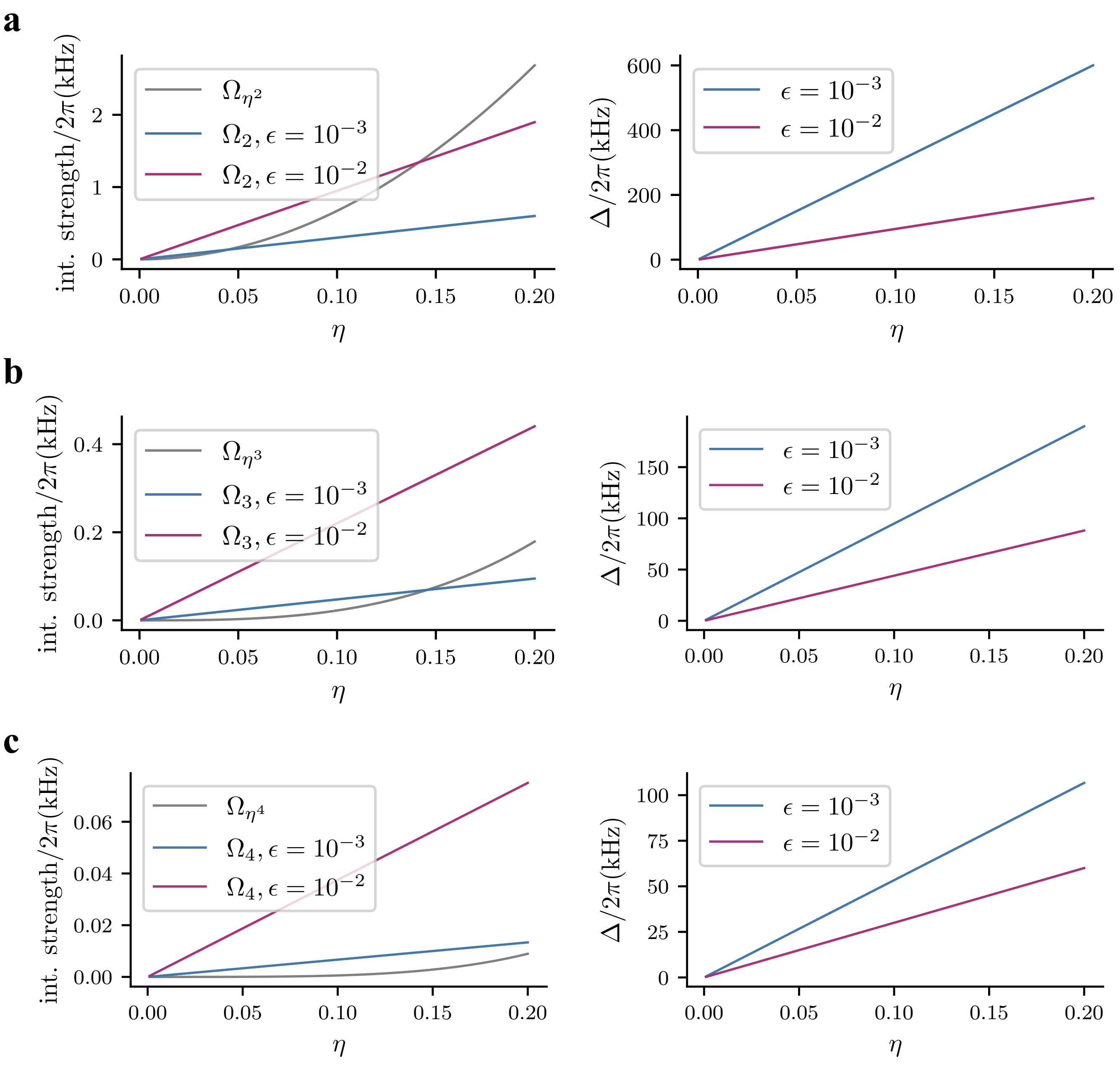

FIG. S.1. Nonlinear interaction strength scaling comparison. We compare the method demonstrated in this work, with magnitude Ω_n, to the method of driving higher-order terms in the Lamb-Dicke expansion, with magnitude Ω_{η^n} (left column). We vary the value of the Lamb-Dicke parameter in this process while keeping the intensity of the driving field constant. For the two non-commuting SDFs method, we keep $\varepsilon = (\Omega_\alpha/\Delta)^{n+1}$ constant by varying the detuning Δ as a function of η (right column). We evaluate this for (a) squeezing, (b) trisqueezing, and (c) quadsqueezing interactions.

these spurious contributions fall into two categories: (1) terms which are off-resonant by $\sim\Delta$ with respect to the leading interaction, and (2) terms which are resonant with the leading interaction.

Similar to the treatment of the off-resonant displacement, which vanishes at integer multiples of $2\pi/\Delta$, the other off-resonant spurious terms can also be suppressed by choosing interaction durations that are integer multiples of $2\pi/\Delta$. In Sec. II B, we further show that this timing constraint can be relaxed by amplitude shaping the pulses over a duration $2\pi/\Delta$, which reduces the contributions of these off-resonant terms through temporal filtering.

In contrast, the spurious terms that are on resonance with the main interaction cannot be eliminated through interaction duration or amplitude shaping. These terms exclusively arise from higher-order contributions in the Magnus expansion. For

a desired interaction of order n, they lead to an error ε in the resulting unitary evolution which scales as $(\Omega_\alpha/\Delta)^{n+1}$, assuming the two SDFs have balanced strengths ($\Omega_\alpha = \Omega_{\alpha'}$). This error decreases faster with Δ than the desired leading interaction. As such, the error can be minimised arbitrarily by increasing Δ.

Looking at this error term in more detail provides further insight into the scaling of the strength of the desired interactions. We assume that errors from off-resonant terms vanish by choosing the interaction duration as an integer multiple of $2\pi/\Delta$. Then, the only remaining error arises from resonant terms. If we accept a fixed error $\varepsilon = (\Omega_\alpha/\Delta)^{n+1} = \text{const.}$ due to these terms, we can express the interaction strength as $\Omega_\alpha = \Delta \sqrt[n+1]{\varepsilon}$. We find that since Ω_α is linear in η, the Lamb-Dicke parameter, Δ also effectively scales linearly with respect to η. When examining the strength of the desired interaction, $\propto \Omega_{\alpha'}\Omega_\alpha^{n-1}/\Delta^{n-1}$, it follows that this quantity can be made effectively linear in η irrespective of n. This tunable scaling is in contrast to driving higher-order spatial derivatives of the field, for which the strength varies as η^n (see Sec. VII).

We emphasize that while the formal expression for Ω_n includes higher powers of η, our scheme allows this dependence to be effectively reduced to linear in η through experimental control of Δ.

To understand this better, we consider two error values $\varepsilon = 10^{-3}$ and $\varepsilon = 10^{-2}$. By fixing $\Omega_\alpha/2\pi = 95\,\text{kHz}$, a value that we can access experimentally, we vary η and adjust detuning Δ in order to keep the error values constant, respectively. As a result, we obtain that the interaction strengths for squeezing Fig. S.1**a**, trisqueezing Fig. S.1**b**, and quadsqueezing Fig. S.1**c** effectively scale linearly with η. We compare these strengths to the interaction strengths due to driving higher order sidebands in the Lamb-Dicke expansion. We assume the same power is available for both methods, i.e., if the two non-commuting SDFs method uses 0.5 mW laser power for each SDF, then the higher motional sideband method, which only requires one bichromatic field, uses 2×0.5 mW laser power.

With the increase in order, the interaction strength decreases for both methods. However, in the case of using two non-commuting SDFs, as the interaction effectively remains linear in η, this shows more favourable scaling, especially with the increase in order. The aim of this analysis is to provide deeper insights into the scaling of the method we introduce. Therefore, in this analysis, we intentionally assume that driving higher-order terms in the Lamb-Dicke expansion yields error-free nonlinear interactions, and we neglect, for example, the effect of spectator modes or off-resonant terms, which can become significant as the Lamb-Dicke parameter η increases, or resonant terms arising from other higher-order contributions in the expansion. Additionally, ε does not represent the error expected in creating any generalised squeezed state; a proper evaluation would require considering the duration of the applied interaction. To address this, in Sec. VIII we discuss in more detail how we evaluate the infidelity $(1-F)$ between the ideal and effective Hamiltonians in simulation using the experimental parameters. Using the interaction strengths Ω_α, $\Omega_{\alpha'}$, and detuning Δ used for generating the states shown in Fig. 3, we obtain infidelities $1-F < 9\times10^{-4}$ for all three cases and we infer the corresponding $\varepsilon \approx 8\times10^{-4}$ for squeezing ($n=2$), $\varepsilon \approx 5.3\times10^{-4}$ for trisqueezing ($n=3$), and $\varepsilon \approx 1.2\times10^{-3}$ and for quadqueezing ($n=4$).

B. Generating the spin-dependent forces

We create the two SDFs required using a Mølmer-Sørensen scheme. Each SDF (as defined in Eq. (4)) requires a bichromatic field which is composed of two tones that are symmetrically detuned from the qubit transition ω_{qubit} by $\pm\delta$. For example, in the case of the SDF conditioned on $\hat{\sigma}_\alpha$, the two tones have the optical phase ϕ_1 and ϕ_2, respectively at the position of the ion. These define the spin-conditioning of the SDF as $\hat{\sigma}_\alpha = \hat{\sigma}_\phi = \hat{\sigma}_{(\phi_1+\phi_2)/2}$, with $\hat{\sigma}_\phi = \cos\phi\,\hat{\sigma}_x + \sin\phi\,\hat{\sigma}_y$, as well as the SDF motional phase $\phi_\alpha = (\phi_1-\phi_2)/2$. This can be extended for the SDF conditioned on $\hat{\sigma}_{\alpha'}$ as well. The relationships between the tone phases and the resulting spin/motional parameters for both SDFs are summarized in Table I.

SDF	Tone 1 Phase	Tone 2 Phase	Derived Quantities and Experimental Settings
SDF conditioned on $\hat{\sigma}_\alpha$	ϕ_1	ϕ_2	$\hat{\sigma}_\alpha = \hat{\sigma}_{(\phi_1+\phi_2)/2} = \hat{\sigma}_\phi$<br>$\phi_\alpha = (\phi_1-\phi_2)/2 = 0$
SDF conditioned on $\hat{\sigma}_{\alpha'}$	ϕ_3	ϕ_4	$\hat{\sigma}_{\alpha'} = \hat{\sigma}_{(\phi_3+\phi_4)/2} = \hat{\sigma}_{\phi+\Delta\phi}$<br>$\phi_{\alpha'} = (\phi_3-\phi_4)/2$

TABLE I. Phase relationships between bichromatic tone phases and the spin/motional parameters of each SDF.

In the majority of experiments, the SDF conditioned on $\hat{\sigma}_{\alpha'}$ was implemented with $\Delta\phi = \pi/2$, ensuring that the spin operators $\hat{\sigma}_\alpha$ and $\hat{\sigma}_{\alpha'}$ are orthogonal. However, for the experimental data shown in Fig. 2**d**, $\Delta\phi$ was varied.

Besides the spin-dependent force term, the bichromatic field also gives rise to a second spin-flip term that drives the qubit transition off-resonantly, so Eq. (4) is modified to:

$$\hat{H}_{\text{bi}} = \frac{\hbar\Omega_\alpha}{2}\hat{\sigma}_\phi\left(\hat{a}e^{-i(\Delta t+\phi_\alpha)} + \hat{a}^\dagger e^{i(\Delta t+\phi_\alpha)}\right) + \hbar\Omega_c\hat{\sigma}_{\phi-\pi/2}\cos(\delta t), \tag{15}$$

where $\Omega_\alpha = \eta\Omega_c$ and the two terms do not commute. The effect of the non-commuting spin-flip term has been extensively discussed in Refs. 42,68,69. When setting $\delta \approx \omega_{\text{osc}}$, it leads to an effective force in the $\hat{\sigma}_\phi$ basis with the strength modulated by the J_0 and J_2 Bessel functions of the first kind, i.e. $\Omega_\alpha \to \Omega_\alpha|J_0(2\Omega_c/\delta) + J_2(2\Omega_c/\delta)|$ in Eq. (4). For these experiments, we operate in a regime of small Ω_c where $|J_0+J_2| \approx 1$. If instead $\delta \approx \omega_{\text{osc}}/2$, we create an effective spin-dependent force in the $\hat{\sigma}_z$ basis with magnitude $\Omega_\alpha \to \Omega_\alpha|J_1(2\Omega_c/\delta) + J_3(2\Omega_c/\delta)|$ in Eq. (4), where J_1 and J_3 are again Bessel functions of the first kind. We use both of these bichromatic field configurations to create the required interaction SDFs in the desired bases.

II. EXPERIMENTAL CONSIDERATIONS

A. Setup

In Fig. S.2**a**, we show our experimental setup. For creating these interactions, we require that the spin components of the SDFs do not commute, i.e., $[\hat{\sigma}_\alpha, \hat{\sigma}_{\alpha'}] \neq 0$. As discussed in Sec. I B, the basis of the SDF depends on the optical phase of the tones at the position of the ion. In Fig. S.2**a**, phase fluctuations might arise from differential path length changes, such as before the fibre beam splitter. We actively stabilise the optical phase, and hence the relative phase between spin components of the two SDFs, $(\phi_3+\phi_4)/2-(\phi_1+\phi_2)/2=\pi/2$, see Sec. I B, to ensure that the non-commutativity relationship is maintained throughout the experiment. The feedback loop relies on measuring the optical interference of the two beams with a photodiode (PD). We measure this interference by sampling a small fraction of light from the two beams using a pick-off mirror (PW). More details on the phase-stabilisation scheme can be found in the Supplemental Material of Ref. 69. Synthesizing these nonlinear interactions does not require $B_0 = 146$ G; this value has been used purely for historical reasons[41].

In Fig. S.2**b**, we show the frequency configuration for implementing the two non-commuting SDFs in the case of squeezing, trisqueezing and quadsqueezing. For squeezing and quadsqueezing, the basis of the two SDFs are $\hat{\sigma}_\phi$ and $\hat{\sigma}_{\phi+\pi/2}$, respectively. To achieve this, the two tones of each of the bichromatic field are symmetrically detuned by $\delta \approx \omega_{\text{osc}}$. For squeezing the exact detuning is $\delta = \omega_{\text{osc}}+\Delta$ for the SDF conditioned on $\hat{\sigma}_\alpha$ and $\delta = \omega_{\text{osc}}-\Delta$ for $\text{SDF}_{\alpha'}$, while for quadsqueezing $\delta = \omega_{\text{osc}}+\Delta$ and $\delta = \omega_{\text{osc}}-3\Delta$, respectively. For trisqueezing, we set the bichromatic field generating the SDF conditioned on $\hat{\sigma}_{\alpha'}$ near resonant with $\omega_{\text{osc}}/2$, with $\delta = (\omega_{\text{osc}}-2\Delta)/2$ such that $\hat{\sigma}_{\alpha'} = \hat{\sigma}_z$. For the SDF conditioned on $\hat{\sigma}_\alpha$, $\delta = \omega_{\text{osc}}+\Delta$ such that its spin basis is $\hat{\sigma}_\phi$.

In Fig. S.2**c**, we show the pulse sequence used for the majority of the data presented in this work. The SDFs conditioned on $\hat{\sigma}_\alpha$ and $\hat{\sigma}_{\alpha'}$ are applied simultaneously to generate the nonlinear interaction, as indicated by the grey dashed box. As shown in Fig. S.3, one can directly measure $P_{|\downarrow\rangle}$ immediately after the interaction without applying any probe pulse. Alternatively, a probe pulse can be used to characterize the resulting motional state. This probe can take the form of an additional SDF or a blue sideband (bsb) pulse. For the data presented in Fig. 2, 3, S.4, S.5, S.7, S.9, and S.11, an SDF probe is used. In Fig. S.6, a bsb probe pulse is employed instead.

B. Ramp

In the experiment, we smoothly ramp the amplitude of the interaction SDF lasers over durations t_{ramp} that are long compared to $2\pi/\Delta$. The amplitude profile of the pulse $g(t)$ is given by

$$g(t) = \begin{cases} \sin^2\left(\frac{\pi t}{2t_{\text{ramp}}}\right),\ t < t_{\text{ramp}} \\ 1,\ t_{\text{ramp}} \leq t \leq t_f - t_{\text{ramp}} \\ \sin^2\left(\frac{\pi(t_f-t)}{2t_{\text{ramp}}}\right),\ t_f - t_{\text{ramp}} < t < t_f. \end{cases} \tag{16}$$

This amplitude shaping serves multiple purposes. While it does help suppress the spin-flip terms, off-resonant by ω_{osc} (see Eq. (15)), the primary motivation is to mitigate the contribution from undesired off-resonant terms in the Magnus expansion. These include the first-order displacement terms and other off-resonant contributions, which are typically detuned by $\sim\Delta$. As discussed in Sec. I A, these terms could be eliminated by precisely setting the interaction durations to integer multiples of $2\pi/\Delta$. However, this duration might change due to offsets in qubit or motional mode frequency. Amplitude shaping offers a more robust solution by reducing the pulse bandwidth in the frequency domain, thereby suppressing the off-resonant driving of unwanted terms in the Magnus expansion. Specifically, choosing $t_{\text{ramp}} \gg 2\pi/\Delta$ ensures that the spectral weight near detuned frequencies $\sim\Delta$ is strongly suppressed.

In the case of squeezing, we use $t_{\text{ramp}} = 40\,\mu\text{s}$ for $\Delta/2\pi = 50\,\text{kHz}$ (i.e. ramp longer by a factor of 2) and for $\Delta/2\pi = 100\,\text{kHz}$ (i.e. ramp longer by a factor of 4). For trisqueezing and quadsqueezing, we use $t_{\text{ramp}} = 80\,\mu\text{s}$ for $\Delta/2\pi = 25\,\text{kHz}$ (i.e. ramp longer by a factor of 2). Due to the timescale of the decoherence channels in our system, we use a smaller value of Δ for the trisqueezing and quadsqueezing interactions to increase their strength.

In Fig. S.3, we explore how the effect of the displacement (first-order in the Magnus expansion) on the squeezing interaction is reduced through the use of a larger duration in the ramp. We start in $|\downarrow\rangle$ and apply the squeezing interaction for variable durations t_{sqz} by setting $m=-1$, $\Delta/2\pi = 50\,\text{kHz}$ and $0.5\,\text{mW}$, as before. The population $P_{|\downarrow\rangle}$ is measured directly after the interaction is applied. The squeezing interaction is expected to be in the $\hat{\sigma}_z$ basis, ideally leaving the initial spin state unchanged. In Fig. S.3, we see that depending on the ramp duration, this is not the case. We observe periodic changes in population which indicate a residual displacement of the oscillator state in phase space.

III. NUMERICAL SIMULATIONS

For the simulations presented in the main text and below, we perform numerical integration of the Lindblad master equation under time-dependent Hamiltonians using the QuantumOptics.jl package in Julia[70]. For creating the squeezed, trisqueezed, and quadsqueezed states, we integrate the Hamiltonian in Eq. (5), without making the RWA with respect to ω_{osc}, and also including the spin-flip terms introduced in Eq. (15). Similarly, the probe SDF is simulated by integrating Eq. (15), but without making the RWA with respect to ω_{osc}.

To recover the Fock states populations, one can drive the blue sideband corresponding to the oscillator. This interaction is

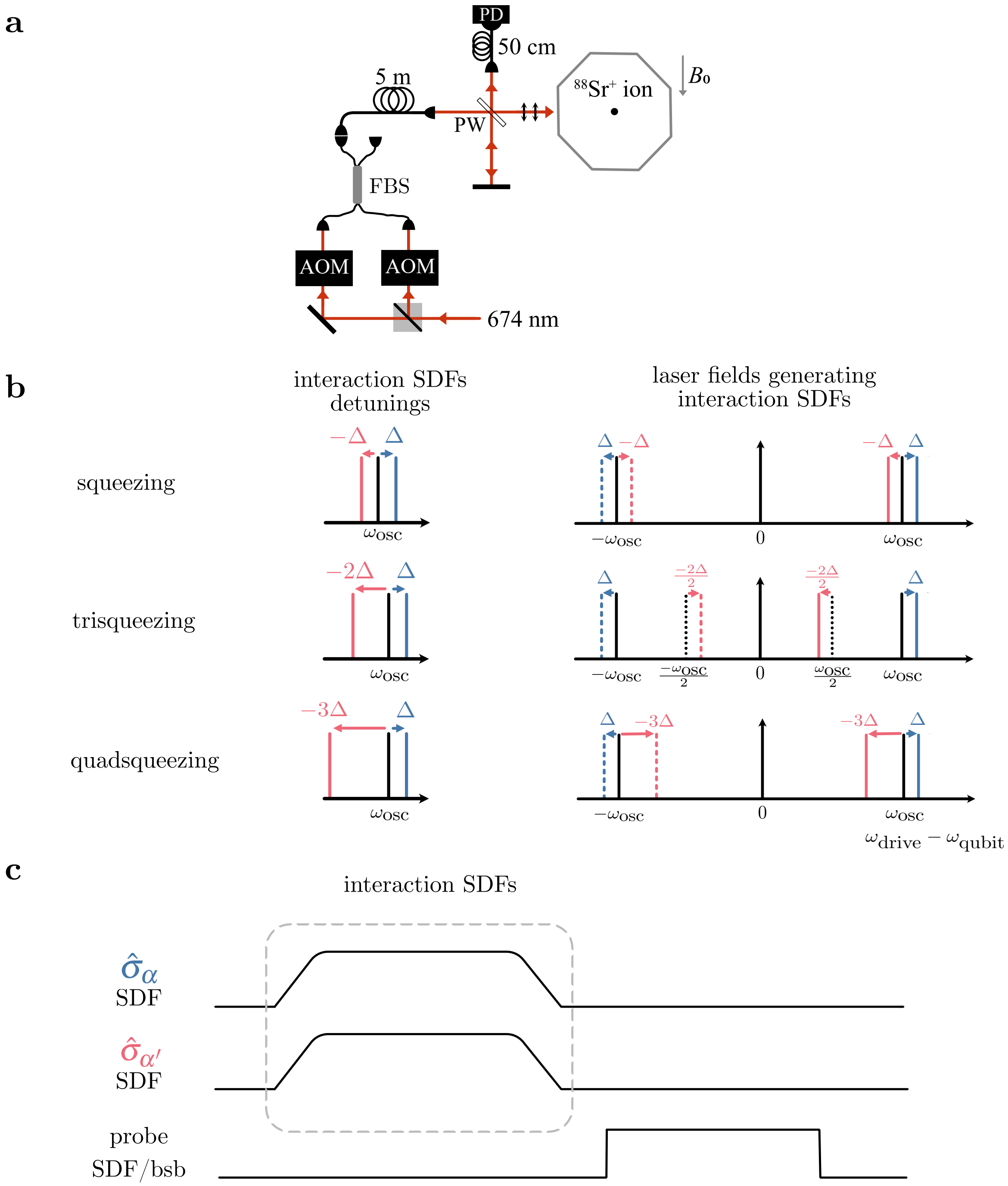

FIG. S.2. Overview of experimental setup. **a** Schematic of the experimental apparatus. The incoming 674 nm beam is split into two beams. The acousto-optic modulators (AOMs) are driven with two radiofrequency tones each to generate two bichromatic fields. The two bichromatic fields are combined using a fibre beam splitter (FBS) and sent to the trap (ion not to scale). We close the resulting interferometer with a pick-off window (PW). We correct for phase fluctuations between the two beams by measuring the interference fringe intensity on a photodiode (PD) and adjusting the phase of the optical field using one of the AOMs. The beam is linearly polarized, as indicated by the black double-headed arrows just before it enters the vacuum chamber (depicted as an octagon). B_0 is the static magnetic field defining the quantization axis. **b** Frequency configuration of the interaction SDFs (left) and the bichromatic laser fields generating them (right). On the left, we show the interaction SDFs: the SDF conditioned on $\hat{\sigma}_\alpha$ (blue) has a fixed detuning Δ from the motional mode frequency $\omega_{\rm osc}$, maintained across all interactions: squeezing, trisqueezing, and quadsqueezing. In red, the SDF conditioned on $\hat{\sigma}_{\alpha'}$ is shown with detunings $-\Delta$, -2Δ, and -3Δ from $\omega_{\rm osc}$ for the respective interactions. On the right, we show the bichromatic laser fields that generate these SDFs. Each bichromatic field consists of two tones symmetrically detuned from the qubit frequency $\omega_{\rm qubit}$: one by $+\delta$ (solid line) and one by $-\delta$ (dashed line). The tones corresponding to the SDF conditioned on $\hat{\sigma}_\alpha$ are indicated in blue, while those for the SDF conditioned on $\hat{\sigma}_{\alpha'}$ are in red. These bichromatic field configurations correspond to the squeezing, trisqueezing, and quadsqueezing interactions, where the detuning of the $\hat{\sigma}_{\alpha'}$ SDF from the motional mode changes as $-\Delta$, $-2\Delta/2$, and -3Δ, respectively. **c** Pulse sequence used for the majority of the data presented in this manuscript (see text).

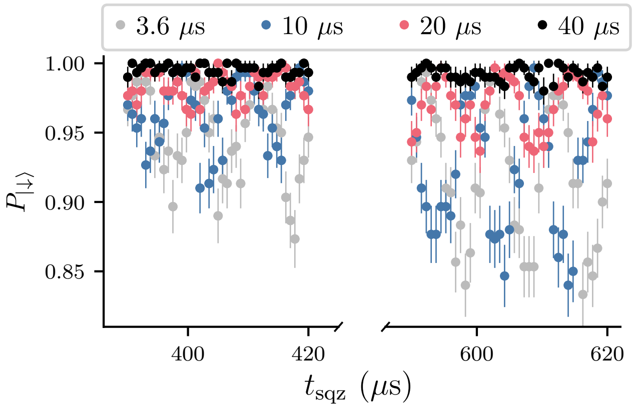

FIG. S.3. Example dynamics from two non-commuting SDFs (Eq. (5)). While these dynamics are measured for the squeezing interaction in the $\hat{\sigma}_z$ basis, they will be similar to any other non-linear bosonic interaction in the same $\hat{\sigma}_z$ basis. The spin state is initially prepared in $|\downarrow\rangle$, before we apply the SDFs for variable durations $t_{\rm sqz}$[46] and measure the probability of staying in the $|\downarrow\rangle$ state, $P_{|\downarrow\rangle}$. We repeat the measurement for different ramp durations. The amount of residual displacement, indicated by a reduction in $P_{|\downarrow\rangle}$, are suppressed as the ramp duration increases. Error bars indicate 68% confidence intervals computed from 300 shots per point; centre equals the measured $P_{|\downarrow\rangle}$.

equivalent to the anti-Jaynes-Cummings Hamiltonian:

$$\hat{H}_{\rm bsb} = \frac{\hbar\eta\Omega_c}{2}(\hat{\sigma}_+\hat{a}^\dagger + \hat{\sigma}_-\hat{a}), \tag{17}$$

which we integrate in simulation in order to analyze the results in Sec. IV.
The motional decoherence (We measured coherence times of 3 ms for the Fock state superposition $(|0\rangle + |1\rangle)/\sqrt{2}$.) in our system is dominated by the heating rate $\dot{\bar{n}}_{\rm osc} = 300\,(20)$ quanta/s, consistent with our simulation results. We introduce the heating by setting the collapse operators[71] to $\sqrt{\dot{\bar{n}}_{\rm osc}}\hat{a}^\dagger$ and $\sqrt{\dot{\bar{n}}_{\rm osc}}\hat{a}$.
The Hilbert space is truncated at phonon number 50 or 150. The higher phonon number is especially necessary when the effect of the probe SDF is simulated.

IV. SQUEEZED STATE CHARACTERISATION

In this section, we discuss how the values for the squeezing parameter r were inferred for the squeezed states as shown in Fig. 2. The ion is initialised to its $|\downarrow\rangle$ state, while its motional state corresponds to a thermal state with $\bar{n}_{\rm osc} = 0.09(1)$ after cooling the motional mode close to the ground state. We then apply the squeezing interaction and the ion is left in the $|\downarrow\rangle|\xi_{\rm th}\rangle$ state, where $|\xi_{\rm th}\rangle$ is the squeezed (parametrised by r and θ, the squeezing axis) thermal state. To determine r, we apply a probe SDF on resonance with the oscillator frequency in the $\hat{\sigma}_x$ basis, for variable durations $t_{\rm probe}$ and power 0.5 mW. As $|\downarrow\rangle = (|+\rangle - |-\rangle)/\sqrt{2}$, where $|\pm\rangle$ are the eigenstates of $\hat{\sigma}_x$, the oscillator state wavefunction is split into two displaced states

$$|\psi\rangle = \frac{1}{\sqrt{2}}(|+\rangle|\alpha,\xi_{\rm th}\rangle - |-\rangle|-\alpha,\xi_{\rm th}\rangle), \tag{18}$$

where $\alpha = -i\eta\Omega_c e^{i\phi_{\rm probe}} t_{\rm probe}/2$ quantifies the displacement. Upon doing a projective measurement in the $\hat{\sigma}_z$ basis, by fluorescence readout, the overlap between the two displaced oscillator states, $f(\alpha,\xi_{\rm th})$ is mapped on the spin. The probability of remaining in $|\downarrow\rangle$ is

$$P_{|\downarrow\rangle} = \frac{1+f(\alpha,\xi_{\rm th})}{2}, \tag{19}$$

where the overlap is defined as[44,72]:

$$\begin{aligned} f(\alpha,\xi_{\rm th}) &= e^{-g(\alpha)h(\xi)}, \\ g(\alpha) &= |2\alpha(t_{\rm probe})|^2\left(\bar{n}_{\rm osc} + \frac{1}{2}\right), \\ h(\xi) &= e^{2r}\cos(\phi_{\rm probe} - \theta/2)^2 + e^{-2r}\sin(\phi_{\rm probe} - \theta/2)^2. \end{aligned} \tag{20}$$

The overlap depends on the relative orientation between the motional phase of the probe SDF $\phi_{\rm probe}$ and the squeezing axis θ. If the two are aligned ($\phi_{\rm probe} - \theta/2 = 0$), the splitting corresponds to the Fig. **2ai** inset. If instead $\phi_{\rm probe} - \theta/2 = \pi/2$, the splitting corresponds to the Fig. **2aiii** inset.
In the experiment, we calibrate the relative orientation $\phi_{\rm probe} - \theta/2$ by keeping the pulse duration for the probe SDF constant while scanning its motional phase $\phi_{\rm probe}$, see Fig. **2c** "start in $|\downarrow\rangle$". We perform a fine scan over one of the peaks and fit a parabola to it in order to determine its centre. Here, the wavefunction is split about the anti-squeezed axis, which we expected to occur for $\phi_{\rm probe}$ an integer multiple of $\pi/2$. However, we observe a phase offset (see Fig. **2c**). We believe that the offset originates from the phase stabilisation. Importantly, the value remains constant over time and does not change with the increase in the pulse duration for the squeezing interaction and can thus be calibrated out. To split about the squeezed axis, we offset the calibrated $\phi_{\rm probe}$ by $\pi/2$.
The model used to fit the experimental data is $P_{|\downarrow\rangle} = (1 + Cf(\alpha,\xi_{\rm th}))/2$, where C accounts for experimental imperfections in the spin state preparation or readout. We first fit the ground-state data by setting $r = 0$ in Eq. (20) and having Ω and C as free parameters, which allows us to extract the strength of the probe SDF. We use this value to fit the splitting about the squeezed axis by setting $\phi_{\rm probe} - \theta/2 = 0$ and having r and C as free parameters.
We observe that heating during the squeezing interaction influences the splitting dynamics, resulting in an overestimate of r, particularly at extended squeezing durations ($t_{\rm sqz} > 400\,\mu$s). Consequently, we attempted to incorporate the heating into our fitting analysis by assuming that the heating and the squeezing interaction are independent processes. Instead of using an initial $\bar{n}_{\rm osc} = \bar{n}_{\rm gs} = 0.09(1)$, we consider that the state before applying the probe was a squeezed thermal state with $\bar{n}_{\rm osc} = \bar{n}_{\rm gs} + \dot{\bar{n}}_{\rm osc} t_{\rm sqz}$.

We verified the validity of this assumption in simulation. We simulated the squeezing interaction followed by applying the probe SDF, see Sec. III. The resulting splitting dynamics were fit in the same way as the experimental data. In Fig. S.4, we compare the results for three squeezing durations, $t_{\rm sqz}$ and two magnitudes parametrised by Δ. In one case, we start with a thermal state with $\bar{n}_{\rm osc} = \bar{n}_{\rm gs} + \dot{\bar{n}}_{\rm osc} t_{\rm sqz}$ and add no heating during the squeezing interaction. In the other case, we start in $\bar{n}_{\rm osc} = \bar{n}_{\rm gs}$ and apply heating during the squeezing interaction. As the squeezing interaction duration is increased, a slight discrepancy in the two models becomes apparent. However, this discrepancy is within the uncertainty of the experimental measurements. We plot the simulation results onto the theory and experimental data, which show good agreement, in Fig. S.4.

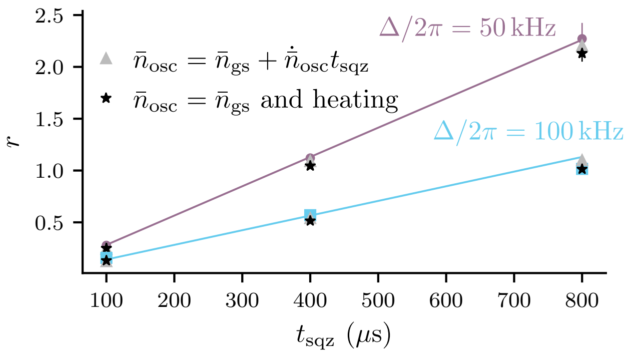

FIG. S.4. Verification of incorporating heating in the fitting model for extracting the squeezing parameter r. We compare the inferred r values for two cases: starting in the ground state with $\bar{n}_{\rm osc} = \bar{n}_{\rm gs}$ and applying the squeezing interaction together with heating (black stars), and starting in a thermal state with $\bar{n}_{\rm osc} = \bar{n}_{\rm gs} + \dot{\bar{n}}_{\rm osc} t_{\rm sqz}$ followed by applying only the squeezing interaction (grey triangles). The second simulated case is equivalent to assuming that the heating and the squeezing interaction are independent. The splitting dynamics for the two cases are simulated and the r is determined by using the same fitting procedure as for the experimental data. We overlay the simulated results to the experimental data and theory lines in Fig. 2**b**. Error bars indicate 68% confidence intervals derived from the fit; centre equals the fitted r.

The heating that occurs when the probe SDF is applied was not included in the fitting analysis. The splitting takes on average around 100 µs and heating effects are negligible when splitting along the squeezing axis.

In Fig. 2**b**, we observe that the for $\Delta/2\pi = 50\,\mathrm{kHz}$, for $t_{\rm sqz} = 1000\,\mu\mathrm{s}$, the inferred r of above 2 has an error bar larger than the other experimental points. Here, the fitting model does not properly describe the splitting dynamics. In Fig. S.5, we show the analysed experimental data for $t_{\rm sqz} = 1000\,\mu\mathrm{s}$ including the fitting and simulation. The oscillations are predicted by the simulation and they are due to the higher-order terms in the Magnus expansion becoming significant. Their effect can be mitigated by increasing Δ, which would reduce the overall strength of $\Omega_\alpha, \Omega_{\alpha'}$ are not adjusted accordingly.

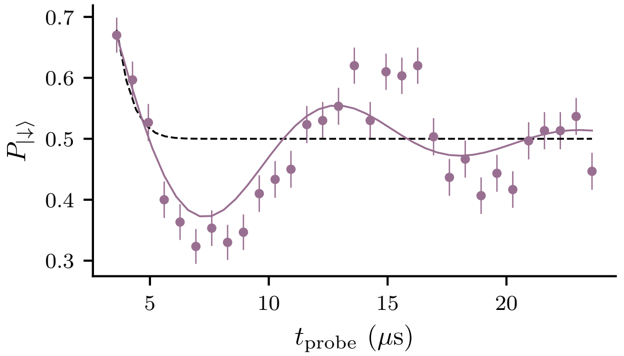

FIG. S.5. Splitting dynamics for applying the squeezing Hamiltonian for $t_{\rm sqz} = 1000\,\mu\mathrm{s}$ with $\Delta/2\pi = 50\,\mathrm{kHz}$ and 0.5 mW for each SDF. We show the fitting to the data using the model described above (dashed black line) and the free parameter simulation (continuous purple line). Error bars indicate 68% confidence intervals computed from 300 shots per point; centre equals the measured $P_{|\downarrow\rangle}$.

An alternative approach to deduce the r value involves extracting Fock state populations by analysing the dynamics of a sideband interaction. Subsequently, a model is used to fit the Fock state populations from the resulting dynamics and thus infer r, again using a model for the Fock state distribution for a given squeezed state. We did not use this method in our study due to heating which occurs during squeezing, which complicates the resulting Fock state distribution. However, we do extract the Fock state populations and compare them to simulation.

We drive the motional blue sideband and fit the dynamics in Fig. S.6**a** to an unconstrained model[8]. The purple vertical bars in Fig. S.6**b** are the inferred Fock state populations. The grey vertical bars are obtained by simulating the squeezing interaction and including the heating rate the corresponding experimental parameters.

V. UNITARITY OF THE INTERACTIONS

An important aspect of our method that was not presented in the main text is its unitarity. Our interaction is unitary, in contrast to approaches relying on dissipative processes for generating nonlinear interactions[58]. The unitarity enables the interaction to be concatenated or arbitrarily placed within a single circuit, making it suitable for continuous variable quantum computing.

In this section, we experimentally investigate and verify the unitarity of our interactions (Fig. S.7). Specifically, we demonstrate that applying the spin-dependent squeezing interaction $\hat{S}$ followed by its adjoint $\hat{S}^\dagger$ to an initial state $|\uparrow, \bar{n}_{\rm osc}\rangle$ leaves the state unchanged.

For this measurement, we initialise the oscillator close to the ground state, apply two consecutive squeezing pulses, and then apply the probing spin-dependent force as described in Fig. 2**a**. In the case of creating a squeezed state, the probing

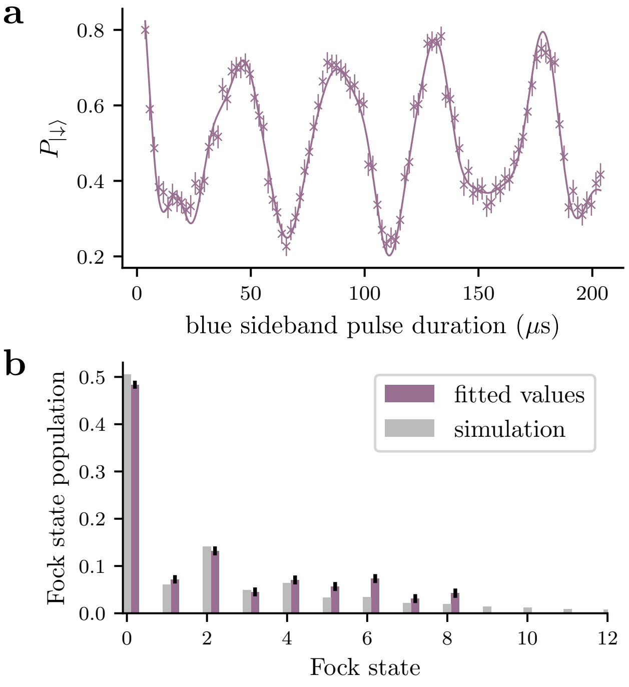

FIG. S.6. Fock state analysis of squeezed states. **a**, Blue sideband dynamics. We apply a blue sideband interaction for a variable duration for a squeezed state with $r = 1.09(4)$. Fock state populations are inferred by fitting an unconstrained model to dynamics. **b**, Fock state population distribution. We plot the histogram of the determined Fock state populations (purple vertical bars) and compare them to simulation (grey vertical bars). Error bars indicate 68% confidence intervals, computed from 300-shot statistics in **a** and derived from the fit used to estimate the Fock-state populations in **b**.

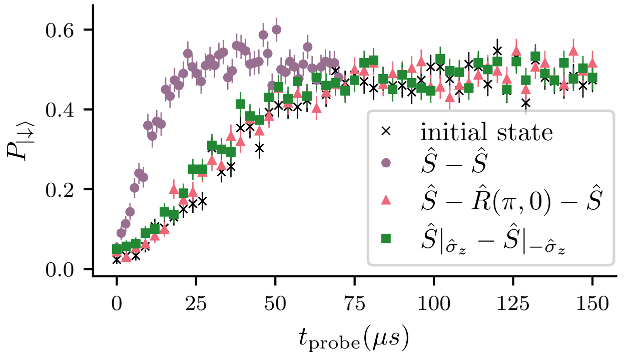

FIG. S.7. Verifying the unitarity of the squeezing interaction. To confirm its unitarity, we use its spin-conditioning property and the control that we have over the basis of the squeezing. We apply a probe SDF for a variable duration and measure the probability of the $|\downarrow\rangle$ state $P_{|\downarrow\rangle}$. We insert a π-pulse on the spin, $\hat{R}(\theta' = \pi, \phi' = 0)$, between two squeezing interactions $\hat{S} - \hat{R}(\pi, 0) - \hat{S}$ (orange triangles) or add a π phase to the spin basis of the second squeezing pulse $\hat{S}|_{\hat{\sigma}_z} - \hat{S}|_{-\hat{\sigma}_z}$ (green squares) and confirm that the resulting splitting dynamics match that of the initial thermal state (black crosses). We also plot the data for two consecutive squeezing pulses (purple circles) which results in a squeezed state. Error bars indicate 68% confidence intervals computed from 300 shots per point; centre equals the measured $P_{|\downarrow\rangle}$.

SDF is always along its short axis. We compared three settings for the two squeezing pulses against the probing of the thermal initial state, where no squeezing pulses are applied.

In the first setting, we apply two identical squeezing pulses for 200 µs each one after the other, i.e. $\hat{S} - \hat{S}$, resulting in a squeezed state. The dynamics of splitting this squeezed state are equivalent to those shown in Fig. 2**a** (purple filled circles), where the squeezing interaction with the same strength is applied for 400 µs. In the second setting, a π-pulse on the spin, $\hat{R}(\theta' = \pi, \phi' = 0)$, is introduced between the two squeezing pulses, $\hat{S} - \hat{R}(\pi, 0) - \hat{S}$. Due to the spin-conditioning of the interaction, the spin-flip induced by the π-pulse transforms the second squeezing interaction into its adjoint. This effectively reverses the effect of the first squeezing interaction and restores the state to the initial thermal state. Note, we initialise the second sequence in $|\downarrow, \bar{n}_{\mathrm{osc}}\rangle$ to keep the readout consistent.

In the third setting, the spin basis of the second squeezing pulse is changed by π by changing the phase of one of the SDFs by π such that the sequence is $\hat{S}|_{\hat{\sigma}_z} - \hat{S}|_{-\hat{\sigma}_z}$. Once again, this transforms the second squeezing interaction into its adjoint, resulting in the final state returning to the initial thermal state.

Each sequence is followed by the probe SDF as explained in Fig. 2**a**.

VI. WIGNER FUNCTION

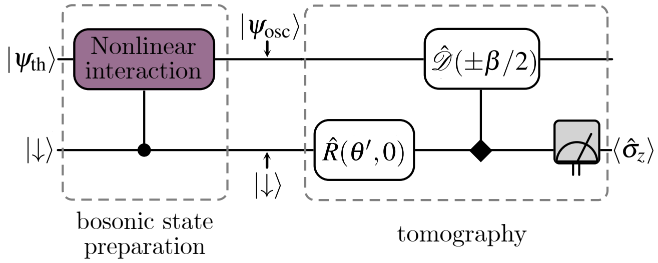

FIG. S.8. Circuit diagram for bosonic state preparation and tomography. The nonlinear interaction, conditioned on $\hat{\sigma}_z$ (circle), is applied to the system initialised in a thermal state and spin down $|\psi_{\mathrm{th}}, \downarrow\rangle$. The characteristic function $\chi(\beta)$ of the resulting oscillator state $|\psi_{\mathrm{osc}}\rangle$ is measured during the tomography step. The rotation $\hat{R}(\theta', \phi' = 0)$ that is applied to $|\downarrow\rangle$ determines if the real or the imaginary part of $\chi(\beta)$ is measured. The displacement is conditioned on $\hat{\sigma}_x$ (diamond), which influences its orientation ($\pm\beta/2$). Finally, the spin state is measured in the $\hat{\sigma}_z$ basis.

The Wigner quasiprobability function $W(\gamma)$[49] describes the wavefunction $|\psi_{\mathrm{osc}}\rangle$ of harmonic oscillator in the position-momentum phase space (x, p) expressed as the complex variable $\gamma = \mathrm{Re}[\gamma] + i\mathrm{Im}[\gamma] \equiv (x + ip)/\sqrt{2}$. The Wigner function fully characterises a state. It is defined as the Fourier transform of the characteristic function $\chi(\beta) = \langle\psi_{\mathrm{osc}}|\hat{\mathscr{D}}(\beta)|\psi_{\mathrm{osc}}\rangle$ with the displacement operator $\hat{\mathscr{D}}(\beta) = e^{\beta\hat{a}^\dagger - \beta^*\hat{a}}$ whose argument

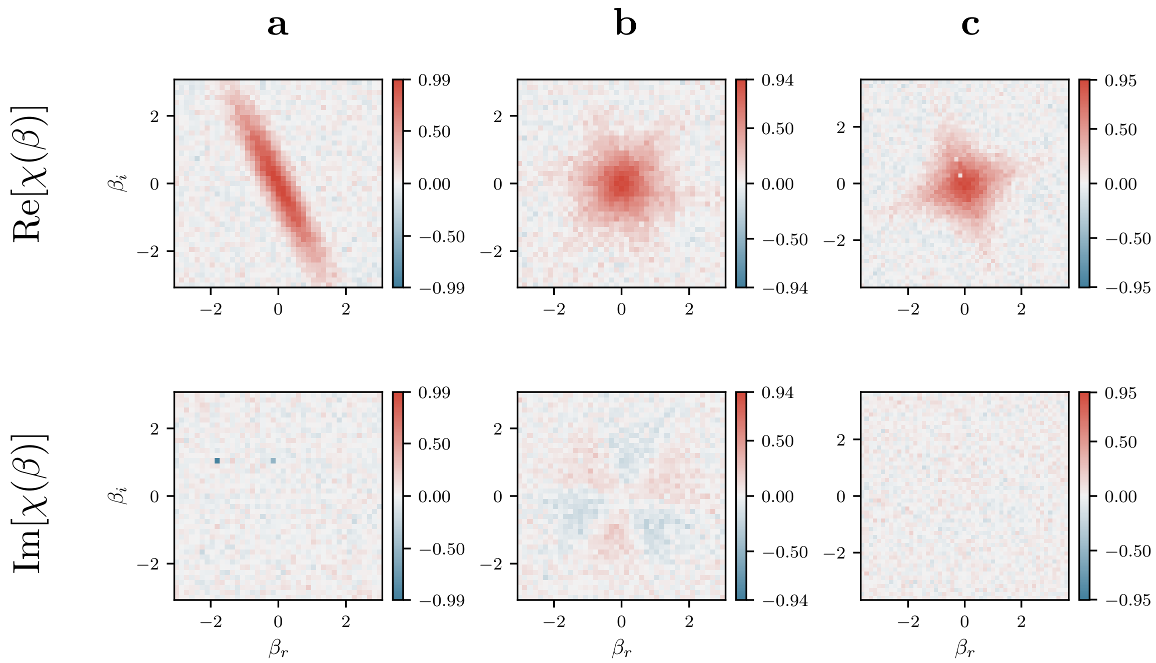

FIG. S.9. Measured characteristic functions for the experimentally realized generalized squeezed states. For each state, we measure the real $\mathrm{Re}[\chi(\beta)]$ and the imaginary part $\mathrm{Im}[\chi(\beta)]$. **a**, For the squeezed state, the imaginary part vanishes. **b**, For the trisqueezed state, the real and imaginary part have a star shaped pattern with six features. The imaginary part oscillates between positive and negative values. The resulting Wigner function has the expected triangular shape. **c**, For the quadsqueezed state, the imaginary part vanishes.

$\beta = \beta_r + i\beta_i$ is the complex displacement variable. Thus,

$$W(\gamma) = \frac{1}{\pi^2}\int \chi(\beta)e^{\gamma\beta^* - \gamma^*\beta} d^2\beta \tag{21}$$

$$\longleftrightarrow W(x,p) = \frac{1}{2\pi^2}\int\int \chi(\beta_r,\beta_i)e^{-2i\frac{x\beta_i - p\beta_r}{\sqrt{2}}} d\beta_r d\beta_i. \tag{22}$$

As demonstrated in Ref. 50,73, the real and imaginary parts of the characteristic function of a harmonic oscillator in a trapped ion system can be measured directly by applying a probe SDF which creates the displacement $\hat{\mathscr{D}}(\beta/2)$ (see Fig. S.8).

For our experiments, after the spin-dependent nonlinear interaction is applied, the system is left in the state $|\psi_{\mathrm{osc}},\downarrow\rangle$. The real part of the characteristic function $\mathrm{Re}[\chi(\beta)]$ is inferred by omitting the single-qubit rotation and directly applying the SDF conditioned on $\hat{\sigma}_x$, i.e. setting $\theta' = 0$ for the single-qubit rotation $\hat{R}(\theta' = 0, \phi' = 0)$ and then, measuring in the $\hat{\sigma}_z$ basis. For the imaginary part $\mathrm{Im}[\chi(\beta)]$, we rotate the spin state in an eigenstate of $\hat{\sigma}_y$ with the rotation $\hat{R}(\theta' = \pi/2, \phi' = 0)$ and then apply the SDF followed by a measurement in $\hat{\sigma}_z$. If the force is applied for a duration t_{probe}, $\beta/2 = \alpha = -i\eta\Omega_c e^{i\phi_{\mathrm{probe}}} t_{\mathrm{probe}}/2$ where ϕ_{probe} denotes the motional phase of the SDF and $\eta\Omega_c$ its strength. We vary both t_{probe} and ϕ_{probe} to sample β in the complex plane. The experimentally measured characteristic functions used to reconstruct the Wigner functions in the main text are shown in Fig. S.9.

We measure 41 settings for each of $\mathrm{Re}[\chi(\beta)]$ and $\mathrm{Im}[\chi(\beta)]$, which are combined to get $\chi(\beta)$. To approximate the Fourier transform of $\chi(\beta)$, we zero-pad the measured data by 200 points on every side resulting in a grid size of 441×441 and perform a discrete Fourier transform. We do not account for the bias parameter that corresponds to state preparation and measurement errors in the spin state. We measure the entire extent of the characteristic function without making assumptions about its hermiticity, as opposed to only measuring half of the complex plane. Our reconstruction technique could also be improved by employing maximum-likelihood estimation, either directly on $\chi(\beta)$ or the Wigner function[73].

VII. SCALING OF THE NONLINEAR INTERACTION STRENGTH

The implementation of squeezing, trisqueezing, and quadsqueezing interactions could also be driven by higher-order terms in the Lamb-Dicke expansion[74] i.e. higher-order spatial derivatives in the field. In Eq. (15) we only consider the first-order term in the Lamb-Dicke expansion. However, higher-order terms are present which can drive higher order motional sidebands. Thus, by using two tones to create a bichromatic field, one can obtain the generalised squeezing interactions. For example, this method has been used to implement squeezing in Ref. 27. The magnitudes of these interactions Ω_{η^n}, where n is, as before, the order of the interaction, are

$$\Omega_{\eta^2,\eta^3,\eta^4} = \left\{\frac{\Omega_c\eta^2}{2!}, \frac{\Omega_c\eta^3}{3!}, \frac{\Omega_c\eta^4}{4!}\right\}. \tag{23}$$

We want to compare these magnitudes to those used to generate the generalised squeezed states in Fig. 3. We assume the

same power is available for both methods, i.e., if the two non-commuting SDFs method uses 0.5 mW for each SDF, then the higher motional sideband method, which only requires one bichromatic field, uses $2 \times 0.5\,\mathrm{mW} = 1\,\mathrm{mW}$.

In Fig. S.10, we plot the ratio of Ω_n and Ω_{η^n} (Eq. (13) and (23)) for the parameters (detuning Δ, power) used to generate the states in Fig. 3. The detuning Δ can be adjusted to increase the magnitude Ω_n. Hence, this plot does not give a comprehensive view of the favorable scaling for the method demonstrated in this paper; see Fig. S.1 instead. However, it shows that, using the same power, our trisqueezing and quadsqueezing interactions are more than 10 and a 100 times stronger, respectively, than interactions using the higher-order derivatives of the field. Without this increase in strength, these interactions would have been unfeasible in our system due to the decoherence effects.

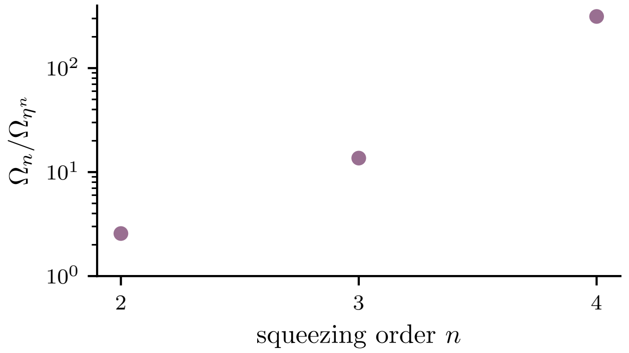

FIG. S.10. Comparison of the strength of generalised squeezing interactions. We compare the method demonstrated in this work, with magnitude Ω_n, to the method of driving higher-order spatial derivatives in the field, magnitude Ω_{η^n}. This comparison is done specifically for the parameters used to generate the states in Fig. 3. We assume the same total amount of laser power is used in both cases.

VIII. COMPARISON OF IDEAL AND EFFECTIVE GENERALISED SQUEEZING INTERACTIONS

We want to evaluate, in simulation, how a state created using Eq. (5), with the carrier included (Eq. (15)) compares to a generalized squeezed state created using the ideal interaction shown in Eq. (11). We consider the overlap between two states given by

$$F(\rho,\sigma) = \left(\mathrm{tr}\sqrt{\sqrt{\rho}\sigma\sqrt{\rho}}\right)^2, \tag{24}$$

where ρ and σ are the density matrices of the two quantum states, respectively. For simulating the states using two non-commuting SDFs, we use the independently measured parameters employed to generate the states in Fig. 3. We then optimize the strength of the ideal generalised squeezing interaction to maximise the overlap between the states. We first consider the case where the oscillator is initialised to its ground state, and that there is no heating during the interaction. Here, $1-F < 9\times10^{-4}$, while for starting in $\bar{n}_{\mathrm{osc}} = 0.09$ and including $\dot{\bar{n}}_{\mathrm{osc}} = 300$ quanta/s during the interactions, $1-F < 1.6\times10^{-3}$. These values apply to the squeezed, trisqueezed, and quadsqueezed states.

IX. QUADSQUEEZED STATE WITH WIGNER NEGATIVITY

We prepare a quadsqueezed state using the same sequence and SDF basis setting as in the main text. The parameters $\Delta/2\pi = 25\,\mathrm{kHz}$ and $t_{\mathrm{ramp}} = 80\,\mu\mathrm{s}$ are unchanged, but we increase the power per interaction SDF to 2 mW and shorten the interaction duration to 400 μs. We obtain the state in Fig. S.11, which shows Wigner negativity. The inferred infidelity is $1-F < 4\times10^{-2}$ ($\varepsilon \approx 7\times10^{-3}$), using the same methodology as the previous section. This result applies for both when initialising in the ground state and having no heating during the interactions and when starting in $\bar{n}_{\mathrm{osc}} = 0.09$ and including $\dot{\bar{n}}_{\mathrm{osc}} = 300$ quanta/s during the interactions. Due to this higher infidelity, we chose not to show this data in the main text. However, we wanted to emphasise that we can find a parameter regime in which we can observe Wigner negativity irrespective of the initial thermal state (impure) and heating rate. In principle, the fidelity can be improved further by tuning the relative ratio between $\Omega_{\alpha,\alpha'}$ and Δ and allowing for a longer duration t_{sqz}.

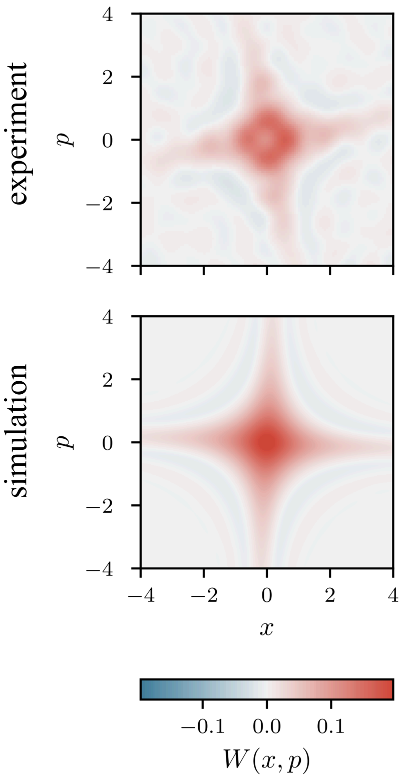

FIG. S.11. Wigner functions of a quadsqueezed state showing Wigner negativity. On the top row, we show the Wigner function $W(x,p)$ reconstructed from experimental data, where x, p are the position and momentum variables associated with the dimensionless position and momentum operators $\hat{x}, \hat{p}$, respectively. On the bottom row, we show the Wigner function of the numerically simulated state with independently measured experimental parameters.

[1] M. M. Fejer, Nonlinear Optical Frequency Conversion, Phys. Today **47**, 25 (1994).

[2] N. Bloembergen, Nonlinear optics and spectroscopy, Rev. Mod. Phys. **54**, 685 (1982).

[3] P. G. Kwiat, K. Mattle, H. Weinfurter, A. Zeilinger, A. V. Sergienko, and Y. Shih, New High-Intensity Source of Polarization-Entangled Photon Pairs, Phys. Rev. Lett. **75**, 4337 (1995).

[4] D. F. Walls, Squeezed states of light, Nature **306**, 141 (1983).

[5] C. M. Caves, Quantum-mechanical noise in an interferometer, Phys. Rev. D **23**, 1693 (1981).

[6] The LIGO Scientific Collaboration, Enhanced sensitivity of the LIGO gravitational wave detector by using squeezed states of light, Nat. Photonics **7**, 613 (2013).

[7] C. A. Casacio, L. S. Madsen, A. Terrasson, M. Waleed, K. Barnscheidt, B. Hage, M. A. Taylor, and W. P. Bowen, Quantum-enhanced nonlinear microscopy, Nature **594**, 201 (2021).

[8] S. C. Burd, R. Srinivas, J. J. Bollinger, A. C. Wilson, D. J. Wineland, D. Leibfried, D. H. Slichter, and D. T. C. Allcock, Quantum amplification of mechanical oscillator motion, Science **364**, 1163 (2019).

[9] A. Mari and J. Eisert, Positive Wigner Functions Render Classical Simulation of Quantum Computation Efficient, Phys. Rev. Lett. **109**, 230503 (2012).

[10] A. Bermudez, G. Aarts, and M. Müller, Quantum Sensors for the Generating Functional of Interacting Quantum Field Theories, Phys. Rev. X **7**, 041012 (2017).

[11] O. Băzăvan, S. Saner, E. Tirrito, G. Araneda, R. Srinivas, and A. Bermudez, Synthetic $\mathbb{Z}_2$ gauge theories based on parametric excitations of trapped ions, Commun. Phys. **7**, 229 (2024).

[12] E. Crane, K. C. Smith, T. Tomesh, A. Eickbusch, J. M. Martyn, S. Kühn, L. Funcke, M. A. DeMarco, I. L. Chuang, N. Wiebe, A. Schuckert, and S. M. Girvin, Hybrid Oscillator-Qubit Quantum Processors: Simulating Fermions, Bosons, and Gauge Fields, arXiv preprint arXiv:2409.03747 (2024).

[13] S. L. Braunstein and R. I. McLachlan, Generalized squeezing, Phys. Rev. A **35**, 1659 (1987).

[14] S. Lloyd and S. L. Braunstein, Quantum computation over continuous variables, Phys. Rev. Lett. **82**, 1784 (1999).

[15] D. Gottesman, A. Kitaev, and J. Preskill, Encoding a qubit in an oscillator, Phys. Rev. A **64**, 012310 (2001).

[16] S. L. Braunstein and P. van Loock, Quantum information with continuous variables, Rev. Mod. Phys. **77**, 513 (2005).

[17] B. De Neeve, T.-L. Nguyen, T. Behrle, and J. P. Home, Error correction of a logical grid state qubit by dissipative pumping, Nat. Phys. **18**, 296 (2022).

[18] V. Sivak, A. Eickbusch, B. Royer, S. Singh, I. Tsioutsios, S. Ganjam, A. Miano, B. Brock, A. Ding, L. Frunzio, S. M. Girvin, R. J. Schoelkopf, and M. H. Devoret, Real-time quantum error correction beyond break-even, Nature **616**, 50 (2023).

[19] K. Banaszek and P. L. Knight, Quantum interference in three-photon down-conversion, Phys. Rev. A **55**, 2368 (1997).

[20] F. Albarelli, M. G. Genoni, M. G. A. Paris, and A. Ferraro, Resource theory of quantum non-Gaussianity and Wigner negativity, Phys. Rev. A **98**, 052350 (2018).

[21] R. Takagi and Q. Zhuang, Convex resource theory of non-Gaussianity, Phys. Rev. A **97**, 062337 (2018).

[22] M. Mielenz, H. Kalis, M. Wittemer, F. Hakelberg, U. Warring, R. Schmied, M. Blain, P. Maunz, D. L. Moehring, D. Leibfried, and T. Schaetz, Arrays of individually controlled ions suitable for two-dimensional quantum simulations, Nat. Commun. **7**, 11839 (2016).

[23] T. Hillmann, F. Quijandría, G. Johansson, A. Ferraro, S. Gasparinetti, and G. Ferrini, Universal Gate Set for Continuous-Variable Quantum Computation with Microwave Circuits, Phys. Rev. Lett. **125**, 160501 (2020).

[24] N. E. Frattini, U. Vool, S. Shankar, A. Narla, K. M. Sliwa, and M. H. Devoret, 3-wave mixing Josephson dipole element, Appl. Phys. Lett. **110**, 222603 (2017).

[25] R. E. Slusher, L. W. Hollberg, B. Yurke, J. C. Mertz, and J. F. Valley, Observation of Squeezed States Generated by Four-Wave Mixing in an Optical Cavity, Phys. Rev. Lett. **55**, 2409 (1985).

[26] E. E. Wollman, C. U. Lei, A. J. Weinstein, J. Suh, A. Kronwald, F. Marquardt, A. A. Clerk, and K. C. Schwab, Quantum squeezing of motion in a mechanical resonator, Science **349**, 952 (2015).

[27] D. M. Meekhof, C. Monroe, B. E. King, W. M. Itano, and D. J. Wineland, Generation of Nonclassical Motional States of a Trapped Atom, Phys. Rev. Lett. **76**, 1796 (1996).

[28] C. W. S. Chang, C. Sabín, P. Forn-Díaz, F. Quijandría, A. M. Vadiraj, I. Nsanzineza, G. Johansson, and C. M. Wilson, Observation of Three-Photon Spontaneous Parametric Down-Conversion in a Superconducting Parametric Cavity, Phys. Rev. X **10**, 011011 (2020).

[29] A. M. Eriksson, T. Sépulcre, M. Kervinen, T. Hillmann, M. Kudra, S. Dupouy, Y. Lu, M. Khanahmadi, J. Yang, C. C. Moreno, P. Deslsing, and S. Gasparinetti, Universal control of a bosonic mode via drive-activated native cubic interactions, arXiv:2308.15320 (2023).

[30] C. Monroe, D. M. Meekhof, B. E. King, and D. J. Wineland, A "Schrödinger Cat" Superposition State of an Atom, Science **272**, 1131 (1996).

[31] S. Haroche, Nobel Lecture: Controlling photons in a box and exploring the quantum to classical boundary, Rev. Mod. Phys. **85**, 1083 (2013).

[32] A. Blais, R.-S. Huang, A. Wallraff, S. M. Girvin, and R. J. Schoelkopf, Cavity quantum electrodynamics for superconducting electrical circuits: An architecture for quantum computation, Phys. Rev. A **69**, 062320 (2004).

[33] R. E. Evans, M. K. Bhaskar, D. D. Sukachev, C. T. Nguyen, A. Sipahigil, M. J. Burek, B. Machielse, G. H. Zhang, A. S. Zibrov, E. Bielejec, H. Park, M. Lončar, and M. D. Lukin, Photon-mediated interactions between quantum emitters in a diamond nanocavity, Science **362**, 662 (2018).

[34] J. I. Cirac and P. Zoller, Quantum Computations with Cold Trapped Ions, Phys. Rev. Lett. **74**, 4091 (1995).

[35] A. Sørensen and K. Mølmer, Quantum Computation with Ions in Thermal Motion, Phys. Rev. Lett. **82**, 1971 (1999).

[36] A. Sørensen and K. Mølmer, Entanglement and quantum computation with ions in thermal motion, Phys. Rev. A **62**, 022311 (2000).

[37] R. Sutherland and R. Srinivas, Universal hybrid quantum computing in trapped ions, Phys. Rev. A **104**, 032609 (2021).

[38] M. Stobińska, A. S. Villar, and G. Leuchs, Generation of Kerr non-Gaussian motional states of trapped ions, EPL **94**, 54002 (2011).

[39] S. O. Mundhada, A. Grimm, J. Venkatraman, Z. K. Minev, S. Touzard, N. E. Frattini, V. V. Sivak, K. Sliwa, P. Reinhold, S. Shankar, M. Mirrahimi, and M. H. Devoret, Experimental Implementation of a Raman-Assisted Eight-Wave Mixing Process, Phys. Rev. Appl. **12**, 054051 (2019).

[40] , this is true up to a phase redefinition for even n; see Supplementary Information.

[41] K. Thirumalai, *High-fidelity mixed species entanglement of trapped ions*, Ph.D. thesis, University of Oxford (2019).

[42] C. F. Roos, Ion trap quantum gates with amplitude-modulated laser beams, New J. Phys. **10**, 013002 (2008).

[43] O. Băzăvan, S. Saner, M. Minder, A. C. Hughes, R. T. Sutherland, D. M. Lucas, R. Srinivas, and C. J. Ballance, Synthesizing a $\hat{\sigma}_z$ spin-dependent force for optical, metastable, and ground-state trapped-ion qubits, Phys. Rev. A **107**, 022617 (2023).

[44] H.-Y. Lo, D. Kienzler, L. de Clercq, M. Marinelli, V. Negnevitsky, B. C. Keitch, and J. P. Home, Spin–motion entanglement and state diagnosis with squeezed oscillator wavepackets, Nature **521**, 336 (2015).

[45] A. I. Lvovsky, Squeezed Light, in *Photonics* (John Wiley & Sons, Ltd, 2015) Chap. 5, pp. 121–163.

[46] , all pulse durations quoted in this text are measured at full-width half-maximum of the pulse shape. The ramp shape is a $\sin(\pi t/2t_{\text{ramp}})^2$ with a total rise time given by the ramp duration t_{ramp}.

[47] D. J. Heinzen and D. J. Wineland, Quantum-limited cooling and detection of radio-frequency oscillations by laser-cooled ions, Phys. Rev. A **42**, 2977 (1990).

[48] M. Wittemer, F. Hakelberg, P. Kiefer, J.-P. Schröder, C. Fey, R. Schützhold, U. Warring, and T. Schaetz, Phonon Pair Creation by Inflating Quantum Fluctuations in an Ion Trap, Phys. Rev. Lett. **123**, 180502 (2019).

[49] E. Wigner, On the Quantum Correction For Thermodynamic Equilibrium, Phys. Rev. **40**, 749 (1932).

[50] C. Flühmann and J. P. Home, Direct Characteristic-Function Tomography of Quantum States of the Trapped-Ion Motional Oscillator, Phys. Rev. Lett. **125**, 043602 (2020).

[51] , we choose this negative detuning $\Delta/2\pi = -25\,\text{kHz}$ to avoid off-resonantly driving an interaction corresponding to another motional mode of the ion.

[52] M. Walschaers, Non-Gaussian Quantum States and Where to Find Them, PRX Quantum **2**, 030204 (2021).

[53] K. R. Brown, C. Ospelkaus, Y. Colombe, A. C. Wilson, D. Leibfried, and D. J. Wineland, Coupled quantized mechanical oscillators, Nature **471**, 196 (2011).

[54] H. C. J. Gan, G. Maslennikov, K.-W. Tseng, C. Nguyen, and D. Matsukevich, Hybrid Quantum Computing with Conditional Beam Splitter Gate in Trapped Ion System, Phys. Rev. Lett. **124**, 170502 (2020).

[55] P.-Y. Hou, J. J. Wu, S. D. Erickson, D. C. Cole, G. Zarantonello, A. D. Brandt, S. Geller, A. Kwiatkowski, S. Glancy, E. Knill, *et al.*, Coherent coupling and non-destructive measurement of trapped-ion mechanical oscillators, Nature Physics **20**, 1636 (2024).

[56] J. Metzner, A. Quinn, S. Brudney, I. D. Moore, S. C. Burd, D. J. Wineland, and D. T. C. Allcock, Two-mode squeezing and SU(1,1) interferometry with trapped ions, Phys. Rev. A **110**, 022613 (2024).

[57] S. Ding, G. Maslennikov, R. Hablützel, and D. Matsukevich, Cross-Kerr Nonlinearity for Phonon Counting, Phys. Rev. Lett. **119**, 193602 (2017).

[58] D. Kienzler, H.-Y. Lo, B. Keitch, L. de Clercq, F. Leupold, F. Lindenfelser, M. Marinelli, V. Negnevitsky, and J. P. Home, Quantum harmonic oscillator state synthesis by reservoir engineering, Science **347**, 53 (2015).

[59] C. Flühmann, T. L. Nguyen, M. Marinelli, V. Negnevitsky, K. Mehta, and J. P. Home, Encoding a qubit in a trapped-ion mechanical oscillator, Nature **566**, 513 (2019).

[60] M. Drechsler, M. Belén Farías, N. Freitas, C. T. Schmiegelow, and J. P. Paz, State-dependent motional squeezing of a trapped ion: Proposed method and applications, Phys. Rev. A **101**, 052331 (2020).

[61] O. Katz, L. Feng, A. Risinger, C. Monroe, and M. Cetina, Demonstration of three-and four-body interactions between trapped-ion spins, Nat, Phys. **19**, 1452 (2023).

[62] Y. Liu, S. Singh, K. C. Smith, E. Crane, J. M. Martyn, A. Eickbusch, A. Schuckert, R. D. Li, J. Sinanan-Singh, M. B. Soley, T. Tsunoda, I. L. Chuang, N. Wiebe, and S. M. Girvin, Hybrid oscillator-qubit quantum processors: Instruction set architectures, abstract machine models, and applications, arXiv preprint arXiv:2407.10381 (2024).

[63] C. H. Valahu, V. C. Olaya-Agudelo, R. J. MacDonell, T. Navickas, A. D. Rao, M. J. Millican, J. B. Pérez-Sánchez, J. Yuen-Zhou, M. J. Biercuk, C. Hempel, T. R. Tan, and I. Kassal, Direct observation of geometric-phase interference in dynamics around a conical intersection, Nat. Chem. **15**, 1503 (2023).

[64] J. Whitlow, Z. Jia, Y. Wang, C. Fang, J. Kim, and K. R. Brown, Simulating conical intersections with trapped ions, Nat. Chem. **15**, 1509–1514 (2023).

[65] J. Lee, N. Kang, S.-H. Lee, H. Jeong, L. Jiang, and S.-W. Lee, Fault-Tolerant Quantum Computation by Hybrid Qubits with Bosonic Cat Code and Single Photons, PRX Quantum **5**, 030322 (2024).

[66] W. Magnus, On the exponential solution of differential equations for a linear operator, Commun. Pure Appl. Math. **7**, 649 (1954).

[67] S. Blanes, F. Casas, J. A. Oteo, and J. Ros, The Magnus expansion and some of its applications, Physics Reports **470**, 151 (2009).

[68] R. Sutherland, R. Srinivas, S. C. Burd, D. Leibfried, A. C. Wilson, D. J. Wineland, D. Allcock, D. Slichter, and S. Libby, Versatile laser-free trapped-ion entangling gates, New J. Phys. **21**, 033033 (2019).

[69] S. Saner, O. Băzăvan, M. Minder, P. Drmota, D. J. Webb, G. Araneda, R. Srinivas, D. M. Lucas, and C. J. Ballance, Breaking the Entangling Gate Speed Limit for Trapped-Ion Qubits Using a Phase-Stable Standing Wave, Phys. Rev. Lett. **131**, 220601 (2023).

[70] S. Krämer, D. Plankensteiner, L. Ostermann, and H. Ritsch, QuantumOptics. jl: A Julia framework for simulating open quantum systems, Comput. Phys. Commun. **227**, 109 (2018).

[71] T. P. Harty, *High-fidelity microwave-driven quantum logic in intermediate-field 43Ca+*, Ph.D. thesis, Oxford University, UK (2013).

[72] M. Affolter, W. Ge, B. Bullock, S. C. Burd, K. A. Gilmore, J. F. Lilieholm, A. L. Carter, and J. J. Bollinger, Toward improved quantum simulations and sensing with trapped two-dimensional ion crystals via parametric amplification, Phys. Rev. A **107**, 032425 (2023).

[73] V. G. Matsos, C. H. Valahu, T. Navickas, A. D. Rao, M. J. Millican, X. C. Kolesnikow, M. J. Biercuk, and T. R. Tan, Robust and Deterministic Preparation of Bosonic Logical States in a Trapped Ion, Phys. Rev. Lett. **133**, 050602 (2024).

[74] D. J. Wineland, C. Monroe, W. M. Itano, D. Leibfried, B. E. King, and D. M. Meekhof, Experimental issues in coherent quantum-state manipulation of trapped atomic ions, J. Res. Natl. Inst. Stand. Technol. **103**, 259 (1998).
